# Comparing self‐reported race and genetic ancestry for identifying potential differentially methylated sites in endometrial cancer: insights from African ancestry proportions using machine learning models

**DOI:** 10.1002/1878-0261.70013

**Published:** 2025-03-06

**Authors:** Huma Asif, J. Julie Kim

**Affiliations:** ^1^ Division of Reproductive Science in Medicine, Department of Obstetrics and Gynecology, Robert H. Lurie Cancer Center Northwestern University Chicago IL USA

**Keywords:** ancestry, DNA methylation, endometrial cancer, epigenetics, machine learning, racial disparities

## Abstract

While the incidence of endometrial cancer is increasing among all US women, Black women face higher mortality rates. The reasons for this remain unclear. In this study, whole genome differential methylation analysis, along with state‐of‐the‐art computational methods such as the recursive feature elimination technique and supervised/unsupervised machine learning models, was used to identify 38 epigenetic signature genes (ESGs) and four core‐ESGs (cg19933311: TRPC5; cg09651654: APOBEC1; cg27299712: PLEKHG5; cg03150409: WHSC1) in endometrial tumors from Black and White women, incorporating genetic ancestry estimation. Methylation at two Core‐ESGs, namely APOBEC1 and PLEKHG5, showed statistically significant overall survival differences between the two ancestral groups (Likelihood ratio test; *P* value = 0.006). Moreover, our comprehensive ancestry‐based analysis revealed that tumors from women with high African ancestry exhibited increased hypomethylation compared to those with low African ancestry. These hypomethylated genes were enriched in drug metabolism pathways, indicating a potential link between genetic ancestry, epigenetic modifications, and pharmacogenomic responses. Combining ancestry, race, and disease type may help identify which patient groups will benefit most from these biomarkers for targeted treatments.

## Graphical abstract

Integrating ancestry, differential methylation analysis, and machine learning, we identified robust epigenetic signature genes (ESGs) and Core‐ESGs in Black and White women with endometrial cancer. Core‐ESGs (namely APOBEC1 and PLEKHG5) methylation levels were significantly associated with survival, with tumors from high African ancestry (THA) showing hypomethylation linked to worse overall survival and enrichment in drug metabolism pathways.

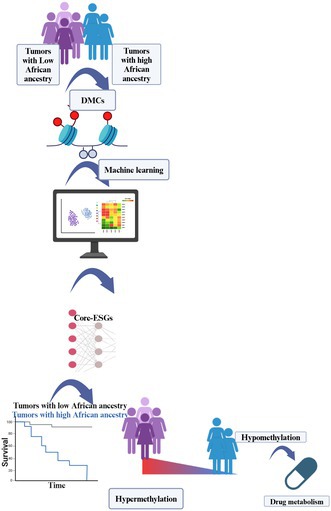

**Abbreviations**
AUC: area under the curve
DMCs: differentially methylated CpGs
TBP: tumors from Black Women
THA: tumors with high African ancestry
TLA: tumors with low African ancestry
TWP: tumors from White women

## 1 Introduction

The molecular details of any given tumor have vast implications for the success or failure of cancer therapies. As sequencing technology rapidly becomes more accessible, the specific epigenetic, genetic and transcriptomic, features of tumors can be used to match the optimal combination, length, and class of therapies for any given cancer type and individual. This is the hope of precision medicine, one that is quickly becoming a reality. Biomarkers are a crucial part of the precision medicine approach, as they provide essential information about an individual's unique biological characteristics, helping to tailor medical treatments and interventions to specific patients. Machine learning (ML), which allows computers to learn from data, can be used to discover biomarkers. ML models retrieve patterns in training data and connect those patterns to classify samples in test data [1]. These models are widely used in the development of biomarkers for treatment outcomes, patient stratification, and drug development, as well as in the identification of biomarkers to predict various types of cancer, including endometrial cancer [2].

Endometrial cancer (EC) is the most common gynecologic cancer in the United States, with an estimated 67 880 new diagnoses and 13 250 deaths expected in 2024 [3]. While most cases occur in postmenopausal women, approximately 20% of endometrial cancers develop in premenopausal women. Important prognostic factors include stage, depth of invasion, cell type, and tumor size [2]. Endometrial cancer (EC) is a heterogeneous disease, classified into less aggressive subtypes, known as Type 1, such as endometrioid adenocarcinoma (stage IA, IB, IIA, IIB) with grade I and II. The aggressive subtypes, known as Type 2, include serous carcinomas and high‐grade endometrioid adenocarcinomas (stage III, IV, and grade III, IV) [4, 5].

In addition to disease‐based heterogeneity, significant racial disparities exist in the risk of EC, with White women showing highest rate of endometrial cancer incidence and Asian women having the lowest rate [6]. Furthermore, it has been observed that Black women experience an 80% higher mortality rate compared to White women, even after adjusting for tumor histology/grade, tumor stage, age, and diagnosis period [7, 8, 9].

Previous studies have reported race‐specific biomarkers due to differences in somatic copy number alteration (sCNA), the number of somatic mutations, and somatic signatures in tumors from Black and White women [10, 11]. Considering the genetic overlap among various racial groups, where there is greater genetic diversity within groups than between them (93–95% vs. 3–5%, respectively) [12, 13] studies exclusively reliant on self‐reported race may not be as reliable and obscure the performance of biomarkers, resulting in a lack of clinical significance. Rather, genetic ancestry, which is more accurately measurable, provides a clearer understanding of the genetic factors involved.

DNA methylation (DNAm), an important epigenetic modification influencing gene regulation, can be influenced by inherited DNA sequence variations as well as a diverse array of environmental factors, including the socioeconomic status, nutrition, and exposure to harmful pollutants [14, 15]. DNAm has been shown to vary between racial groups across different types of cancer, and many studies have utilized differentially methylated CpG sites (DMCs) as biomarkers for cancer detection due to their stability and efficient detection using multiple assays [16, 17, 18, 19]. Our previous work had uncovered racial differences in DNA methylation that revealed endometrial tumors from self‐reported Black women were hypervariable and hypomethylated compared to tumors from White women [20]. Despite previous findings, the complex interplay between genetic ancestry and DNA methylation patterns in endometrial tumors remains poorly understood, as race, being a social construct, does not fully capture the genetic influences involved.

In this study, we used machine learning to identify epigenetic signature genes linked to endometrial cancer in tumors from Black and White patients, considering race and ancestry‐specific changes in DNA methylation patterns. We propose that these signatures can be used to develop prognostic models for personalized treatment and endometrial cancer risk stratification, which can help identify high‐risk individuals and predict recurrence, progression, and response to treatment in diagnosed patients.

## 2 Materials and methods

### 2.1 Sample source for biomarker discovery and data preprocessing

We downloaded clinical and methylation data of 393 endometrial tumor samples (294 White samples and 99 Black samples) from The Cancer Genome Atlas (TCGA) [21] using the TCGABiolinks package (v2.18.0) [22]. The methylation data was produced utilizing the Illumina Infinium Human Methylation 450 Beadchip (450 K array). The methylation levels were quantified as beta values, calculated using the formula Meth/(UnMeth + Meth), wherein ‘Meth’ and ‘UnMeth’ denote the intensities of methylated and unmethylated probes, respectively, on the Illumina 450 K array. To reduce false positives, we excluded probes affected by SNPs (those containing SNPs with a minor allele frequency < 0.05), probes with missing values, and cross‐reactive probes (those that mapped to multiple locations in the genome) [23]. To enhance statistical interpretation, the beta values were transformed into *M* values, calculated as log2 (β/1 − β) [24].

### 2.2 Ancestry information

We extracted the ancestry data of each sample using genomic ancestry calls provided by Carrot‐Zhang et al. [25], employing the computational methods outlined in their research. We combined the samples with ancestry information and methylation data using patient IDs.

Briefly, the global ancestry of each TCGA patient in the Carrot‐Zhang et al. paper [25] was determined using five independent classification methods: Washington University, University of California San Francisco (UCSF), University of Trento, Broad Institute, and the Exome Aggregation Consortium (ExAC) methods, each employing SNP array and/or exome sequencing data.

In the Washington University method, Birdseed genotype files were converted to individual VCF files and merged into combined VCFs. Ancestry estimates were derived using PC1 and PC2 after conducting PCA, implemented by plink 1.9 [26]. In the ucsf method [25], ancestry calls were computed based on partition around medoids (PAM) clustering of principal components (PCs) generated from quality‐controlled genotyping files. In the University of Trento method, ancestry estimates were generated using ethseq [27]. In the Broad Institute method, ancestral groups were defined using the 1000 Genome cohort, and admixture version 1.23 was used to estimate the percentage of global ancestry [28]. The ExAC dataset's ancestry calls were generated through principal component analysis (PCA) of common exome SNPs, which stratified the exomic data into principal components and identified major clusters representing continental ancestry [29]. Finally, consensus ancestry calls were determined based on the ancestral population that had the majority of assignments for each sample using all methods. Tumor patients with a main ancestry proportion below 80% or a secondary ancestry proportion above 20% were classified as admixed [25].

### 2.3 Identification of differential methylated CpGs (DMCs) and pathway enrichment analysis

Differentially methylated CpGs (DMCs) analysis was performed using the Linear Models for Microarray Data (Limma) r package (v3.46.0) in tumors with low African ancestry (TLA) (tumors with less than 80% African ancestry) compared to tumors with high African ancestry (tumors with greater than 80% African ancestry) (THA). Our analysis specifically focuses on tumor samples within each racial/ancestral group. However, due to the limited number of normal samples in each group, tumor vs. normal comparisons were not possible. DMCs with a false discovery rate (FDR) corrected *P* value of 0.01 and an absolute log fold change > 0.5 were extracted [30]. DMCs with average methylation levels greater in TLA than THA were classified as hypermethylated in TLA or hypomethylated in THA. Genome annotation of these DMCs was performed using the Illumina protocol, that is, IlluminaHumanMethylation450kanno.ilmn12. hg19. Functional enrichment (Kyoto Encyclopedia of Genes and Genomes; KEGG) analysis for DMCs was performed using the clusterprofiler package (v.4.2.2), with a false discovery rate threshold of < 0.05.

### 2.4 Correlation of promoter‐associated ESGs with gene expression

We assessed the correlation of promoter‐associated DMCs with gene expression. Initially, we identified promoter‐associated CpGs and calculated the absolute mean methylation differences (delta) between tumors with high and low African ancestry, filtering CpGs with a delta greater than 10% (*n* = 82 CpGs). Subsequently, RNA‐sequencing data, along with clinical information for the selected tumor samples, was obtained from The Cancer Genome Atlas (TCGA) using the tcgabiolinks package (v2.18.0) [22]. Raw count data were normalized to log2‐counts per million (CPM) using the voom function [30]. We conducted differential expression gene (DEGs) analysis comparing TLA vs. THA using the edger‐limma workflow and filtered DEGs with Benjamini–Hochberg (BH) adjusted *P* values < 0.05, resulting in 1853 statistically significant differentially expressed genes. We extracted DEGs showing statistically significant methylation differences (71/82 genes) and conducted Pearson correlation analysis, filtering CpG‐gene pairs with |*r*| > 0.3 and adjusted *P* values < 0.05.

### 2.5 Identification of epigenetic signature genes (ESGs) and Core‐ESGs

The DMCs that showed statistically significant differences between Black and White women with EC (Table S4) were extracted for racial information and methylation values (*M*‐values) for the subsequent classifier model building. The pandas package was imported and categorical race values were one‐hot encoded as binary outcome using the pandas.get_dummies function (https://pandas.pydata.org/pandas‐docs/stable/reference/api/pandas.get_dummies.html). For all machine learning experiments performed in this paper, we used Scikit‐learn library (sklearn) and Python and R programming language (r version 4.1.1). Sklearn use Numpy arrays so both predictors (DMCs) and outcome (Race) variables were converted into Numpy arrays. Data was randomly split into training and test data using ‘train_test_split’ at an approximate ratio of 70: 30. Finally, the variable values were centered and scaled using scikit‐learn's StandardScaler (https://scikit‐learn.org/stable/modules/generated/sklearn.preprocessing.StandardScaler.htm) which subtracts the mean of the values in a variable in the training set and then divides it by the standard deviation of those values. We use two‐step hybrid feature selection technique to reduce the number of DMCs into epigenetic signature genes (ESGs) and Core‐ESGs. In the first step, Recursive feature elimination (RFE) [31, 32] wrapped in binary logistic regression was used for DMCs selection. Model performance was evaluated after every DMC removal and terminated when highest accuracy score was found. Model prediction accuracy was selected by plotting receiver operating characteristic (ROC) curves and highest area under the curve (AUC) values from the results of 10 cross‐validations. To select the final model for our ESGs, binary logistic regression models were fitted to predict race (White/Black) using different combinations of these RFE filtered DMCs values as predictors, with a total of 8000 combinations tried. In the second step, we performed a redundancy filtering based on K‐means clustering in r software, to remove DMCs with similar information. We use both Elbow and Silhouette method for number of clusters (or K) selection. Clusters were visualized using the factoextra r package function fviz_cluster(). We selected 15 closest neighbors using get.knnx() function from FNN r package and final core‐ESGs were selected based on highest logistic regression odds. Binary logistic regression was fitted to predict race using these Core‐ESGs and model performance was evaluated using ROC like the one described above for ESGs. We used drug‐Gene Interaction Database (DGIdb, http://www.dgidb.org) to find potential therapeutic drugs.

### 2.6 Performance of epigenetic signature genes (ESGs) based on disease type

To predict the performance of ESGs based on disease type, we stratified the tumor samples into aggressive and less aggressive subtypes. We classified the tumor samples with primary diagnosis as endometrioid adenocarcinoma (stage IA, IB, IIA, IIB) with grade I and II as less aggressive disease type (Type 1) and those with primary diagnosis as Serous, Adenocarcinomas and endometrioid adenocarcinoma (stage III, IV, and grade III, IV) as aggressive subtype (Type 2). We then fit logistic regression to predict aggressive and less aggressive disease type using different combinations of these RFE filtered 38 DMCs values as predictors. We performed three different analyses where in the first analysis we ran the model independent of race to check how well it performs in separating aggressive and less aggressive subtype. In second and third analysis, we split Black and TWP samples into more aggressive and less aggressive subtypes and evaluated their performance in each group.

### 2.7 Visual performance of ESGs and core‐ESGs using unsupervised hierarchical clustering and t‐SNE

Unsupervised hierarchical clustering of endometrial tumor samples based on ESGs and Core‐ESGs was performed using Euclidean distance and complete linkage method with dist(), hclust() and cutree() functions in r software. The Elbow and Silhouette method was used for number of clusters (or K) selection. Clusters were visualized using the factoextra r package function fviz_cluster() and pheatmap r package. To confirm the clusters identified, t‐SNE analysis was performed using TSNE from sklearn.manifold.

### 2.8 Overall survival analysis of ESGs and core‐ESGs

Overall survival data for each endometrial tumor sample used in the analysis was downloaded from the ucsc xena Browser (https://xenabrowser.net/datapages/). Overall survival was measured in days from the date of diagnosis to either the date of death or the last follow‐up for living samples (Os.time). Tumor samples from patients who died during the follow‐up period were coded as 1 (= dead), while those from patients still alive at their last follow‐up were coded as 0 (= censored) and stored in the variable vital_status. The Cox proportional hazards regression model was used for multivariate and univariate survival analyses. The response variable was a Surv object containing OS.time and vital_status, created using the Surv command. Ancestry proportions (low/high African) and all core‐ESGs were used as predictors.

For univariate analysis, the coxph from survival r package was used to analyze the association of methylation profile of each ESG with patient survival in each tumor sample [33]. Any gene with *P* < 0.05 was considered as survival correlated ESG and can be used as promising prognostic biomarker. For multivariate analysis with core‐ESGs, each predictor in the multivariate model was checked for violations of the proportionality assumption using the function cox.zph from the survival r package.

## 3 Results

### 3.1 Differential methylation analysis of endometrial tumors in black and white women based on ancestry

We analyzed genome‐wide methylation data from a total of 393 endometrial tumor samples. Among these samples, 75% were from self‐reported White women (tumors from White patients – TWP), and 25% were from self‐reported Black women (tumors from Black patients – TBP). The genetic ancestry and self‐reported race were matched in TWP (99% self‐reported TWP, with European ancestry). TBP exhibited a varied proportion of African ancestry and were categorized into two groups: African and African‐admixed, based on their proportion of African ancestry. Tumors with greater than 80% African ancestry were designated as Africans (comprising 65% of self‐reported TBP), while those with an African ancestry proportion less than 80% were classified as African‐admixed (constituting 33% of self‐reported TBP). Patients with African ancestry had a higher percentage of advanced‐stage samples (stage III and IV) compared to those with African‐admix and European ancestry (38.4% African compared to 27.2% African‐admix and 27.9% European). Clinical and ancestry details of samples are given in Table 1.

**Table 1:** Ancestry and clinical features of tumor samples used in the analysis.

Clinical features	European women (%)	African women (%)	African‐admix (%)	Missing_ancestry	Total
BMI	286 (72)	65 (16.5)	33 (8.3)	9	393
<25*	57 (19.9)	4 (6.2)	1 (3.04)		62
25–40**	146 (51)	40 (61.5)	19 (57.57)		205
≥ 40***	69 (24.1)	14 (21.5)	9 (27.27)		92
BMI_not_reported	14 (5)	7 (10.8)	4 (12.12)		25
Race					393
White (294)	284 (99.3)	1 (0.35)	1 (0.35)	9	286
Black (99)	2 (2.02)	64 (64)	32 (33)		98
Age (years)					
≤ 50	23 (8.0)	3 (4.61)	1 (3.0)		27
50–64	123 (43)	29 (44.6)	15 (45.4)		167
> 65	139 (48.6)	33 (50.7)	17 (51.5)		189
Age_not_reported	1	NA	NA		1
FIGO_Stage					
I	181 (63)	31 (47.7)	21 (63.6)		233
II	25 (8.7)	9 (13.8)	3 (9)		37
III	65 (22.7)	22 (33.8)	7 (21.2)		94
VI	15 (5.24)	3 (4.6)	2 (6.0)		20
Histology					
Endometrioid adenocarcinoma Not otherwise specified (NOS)	208 (72)	43 (66)	19		270
Endometrioid adenocarcinoma, secretory variant	2 (0.7)	NA	NA		2
Papillary serous cystadenocarcinoma	4 (1.39)	1 (1.53)	NA		5
Serous cystadenocarcinoma, NOS	72 (25.1)	21 (32.3)	12		105
Carcinoma, undifferentiated, NOS	NA	NA	1		1
Adenocarcinoma, NOS	NA	NA	1		1
Grade					
G1	46	6	6		58
G2	61	17	7		85
G3	170	41	20		231
High	9	1	NA		10

**Fig. 1:**
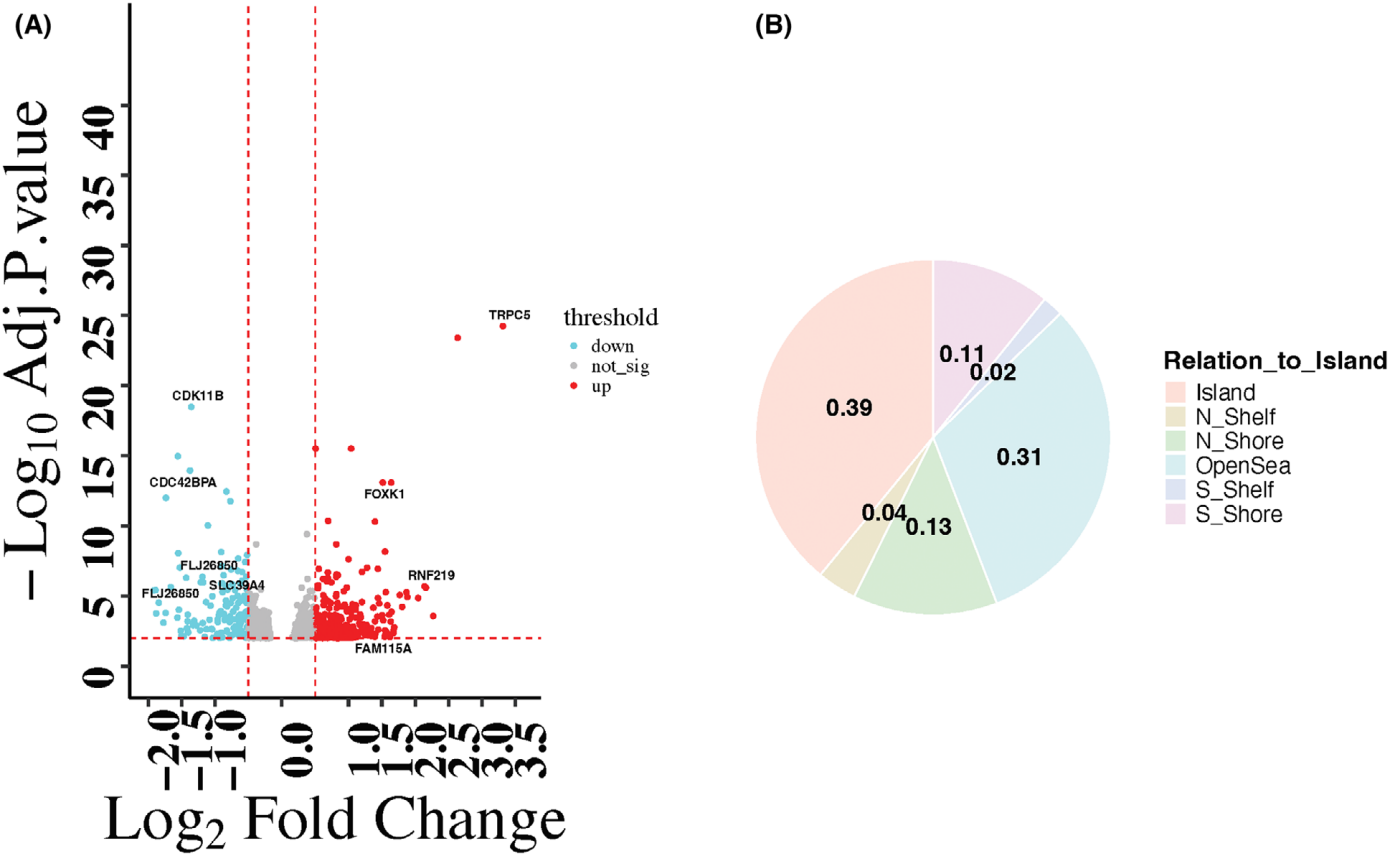
(A) Volcano plot for differentially methylated CpGs (DMCs) in TLA (tumors with less than 80% African ancestry, *n* = 319) versus THA (tumors with greater than 80% African ancestry; *n* = 65). The *x* axis represents the log fold change and *y* axis represent −log10 false discovery rate (FDR) adjusted *P* values. Significance thresholds are shown by red dotted lines (FDR adj *P* value = 0.01 (−log10(0.01) = 2) and absolute log fold change greater than and equal to 0.5). A total of 471 differentially methylated CpGs (DMCs) were identified. Red dots indicate hypermethylated CpGs in TLA compared to THA (total 325) while turquoise dots represent hypermethylated DMCs in THA relative to TLA (total 146). Insignificant CpGs are shown in gray. Overall, THA showed more hypomethylation as compared to TLA (69% in THA vs. 31% in TLA tumor samples). (B) Distribution of DMCs in different genomic regions.

To identify differentially methylated CpGs (DMCs) in our tumor samples based on ancestry, we divided the samples into two groups: high Africans (tumors with greater than 80% African ancestry) and low Africans (tumors with less than 80% African ancestry). Differentially methylated CpGs (DMCs) were identified between these two groups. We identified 471 statistically significant DMCs (*P*adj = 0.01; abs(logFC) > 0.5; Fig. 1A). Most DMCs were hypomethylated in tumors from high African ancestry (69%) compared to tumors with low African ancestry (31%). Approximately 27% of the DMCs had a log fold change (log2FC) greater than 1, indicating that these DMCs had a greater than two‐fold difference in methylation levels between the two ancestral groups. The complete list of DMCs is provided in Table S1. The complete distribution of DMCs in different genomic regions is shown in Fig. 1B.

**Fig. 2:**
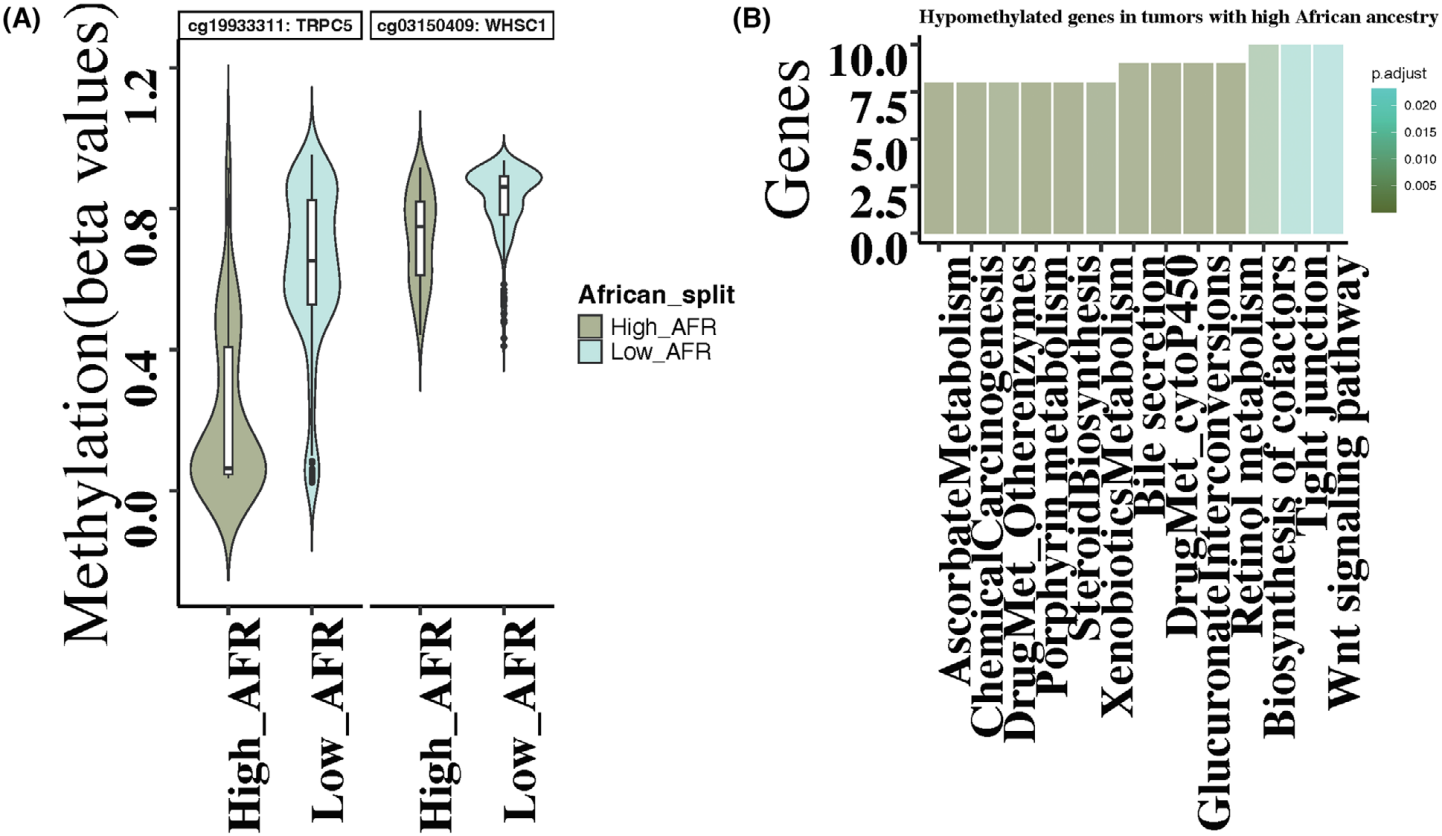
(A) Violin plot of significant differentially methylated CpGs (DMCs) between tumors with high African ancestral proportions (*n* = 65) and tumors with low African ancestral proportions (*n* = 319). CpG sites cg19933311, cg03150409, showed that as African ancestral proportion increases the hypomethylation increases. The line in the center represents average methylation in each group. The whiskers represent the upper and lower quartiles, extending to ±1.5 times the interquartile range (IQR). Turquoise color is showing CpG sites in tumors with African ancestry proportion < 80% i.e., Low_AFR samples and dark olive green is for tumors with African ancestry proportion > 80% i.e., High_AFR. (B) KEGG pathway enrichment analysis of hypomethylated genes in THA. Pathways with FDR < 0.05 are shown. Genes with hypomethylation in tumors from African women are enriched in drug metabolism pathways.

We previously reported 704 DMCs between tumors from Black and White patients based on self‐reported race [20]. We found that 67% of these DMCs were shared between the self‐reported race and ancestry, while 33% were unique to this ancestry‐specific analysis (Fig. S1, Table S2). KEGG pathway analysis of hypomethylated genes in tumors from high African ancestry revealed enrichment in several pathways, including the Wnt signaling pathway, retinol metabolism, drug metabolism‐cytochrome P450, Metabolism of xenobiotics by cytochrome P450, Drug metabolism‐other enzymes, and Chemical carcinogenesis‐DNA adducts (Fig. 2).

In contrast, analysis of hypomethylated genes in tumors from European ancestry revealed enrichment in proteoglycans in cancer, the mTOR signaling pathway, cellular senescence, oocyte meiosis, and the AMPK signaling pathway (Fig. S2A). Notably, none of these drug metabolism‐related pathways identified in this ancestry‐specific analysis appeared in our previous race‐based analysis, emphasizing the importance of integrating ancestry into such analyses.

### 3.2 PCA reveals epigenetic heterogeneity in African‐admixed populations

PCA analysis reveals that African‐admixed samples exhibit diverse methylation profiles. Some samples cluster closer to European samples, while others resemble African samples. Additionally, certain samples form distinct clusters, particularly those with PC1 values between 0.20 and 0.33 and PC2 values below 0 (Fig. S2B). These findings highlight the epigenetic heterogeneity within African‐admixed populations, which is influenced by varying proportions of African and European ancestry. A comparison of methylation beta values between African and African‐admixed individuals shows that the median methylation value is lower in African individuals (0.22) compared to African‐admixed individuals (0.44). However, these differences did not reach statistical significance, likely due to the small sample size (Fig. S2B).

### 3.3 Effects of DNA methylation on gene regulation

To examine the correlation between methylation sites and gene expression, we identified differentially expressed genes (DEGs) in our tumor samples based on ancestry by comparing the high African and low African groups as described for the DMCs analysis. This comparison revealed 1853 statistically significant DEGs (*P*adj < 0.05). Pre‐ranked gene set enrichment analysis (GSEA) of all expressed genes revealed downregulation of genes associated with oxidative phosphorylation, MTORC1 signaling, cholesterol homeostasis, DNA repair, and interferon gamma and alpha responses, while genes linked to hedgehog signaling and KRAS signaling DN (downregulated) were upregulated in tumors from African women. Downregulation of cholesterol homeostasis and MTORC1 pathways, along with upregulation of KRAS signaling DN, were novel findings not observed in our previous race‐based analysis (Fig. S3 and Table S3).

The relationship between methylation and gene expression in tumors from high and low African ancestry was determined by extracting sites with absolute mean methylation differences (delta) greater than 10% within the promoter region. We identified 82 CpG‐gene pairs associated with a delta greater than 10 % (Table S4A). We performed Pearson correlation analysis between the 82 CpG‐gene pairs and the differentially expressed genes. This analysis identified 22 CpG‐gene pairs with a statistically significant correlation between methylation and gene expression values.

Integrating the results from our previous race‐based analysis revealed that the top significantly correlated genes were C20rf43 (LDAH) and LINC00667 consistent with previous report [20]. Additionally, we identified 11 sites that were unique to this analysis (Table S4B). One of the unique ancestry‐specific CpGs (cg13648501) was found to be associated with the NR3C1 gene, which codes for the glucocorticoid receptor protein. This receptor is involved in the human response to environmental stress. This gene is hypomethylated in tumors with high African ancestry.

### 3.4 Machine learning integration of racial and ancestry‐specific differential methylation CpGs (DMCs) in EC

**Fig. 3:**
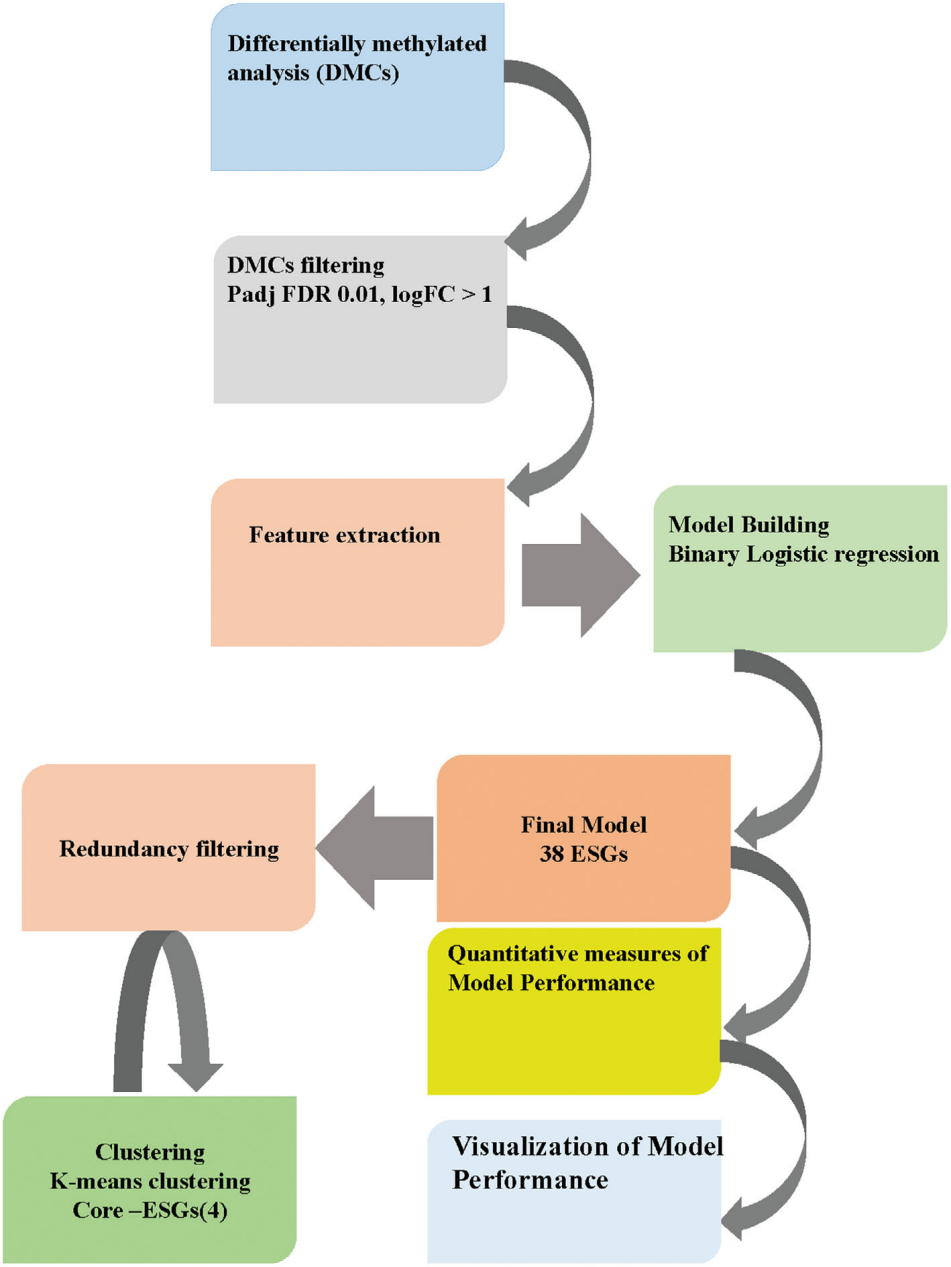
Schematic Diagram Illustrating Epigenetic signature genes (ESGs) and Core–ESGs Identification Methods.

To integrate the differential methylation patterns identified in our previous study focusing on racial differences between Black and White women with endometrial cancer into this ancestry‐based analysis, we employed a machine learning approach (Fig. 3). We utilized the DMCs identified in our previous race‐based analysis as input features for developing a predictive model [20].

Specifically, we trained a logistic regression classifier model using the methylation m‐values of the 704 DMCs obtained from 393 tumor samples, which included 294 samples from self‐reported White women and 99 from TBP samples (Table S5).

### 3.5 Building a predictive multi‐gene epigenetic signature (ESGs) model using hybrid feature selection technique

First, to correctly adjust the predictive model, we split the standardized data into 70/30 training/validation test sets and ran recursive feature elimination program (RFE) of a Scikit‐learn (sklearn) [34]. We ran Scikit‐learn Logistic Regression program with regularization using L2 (Ridge) penalty, and sklearn.metrics for measuring model performance. After RFE, we were left with 38 DMCs (now referred to as epigenetic signature genes (ESGs)). The model was validated using tenfold cross‐validation.

**Fig. 4:**
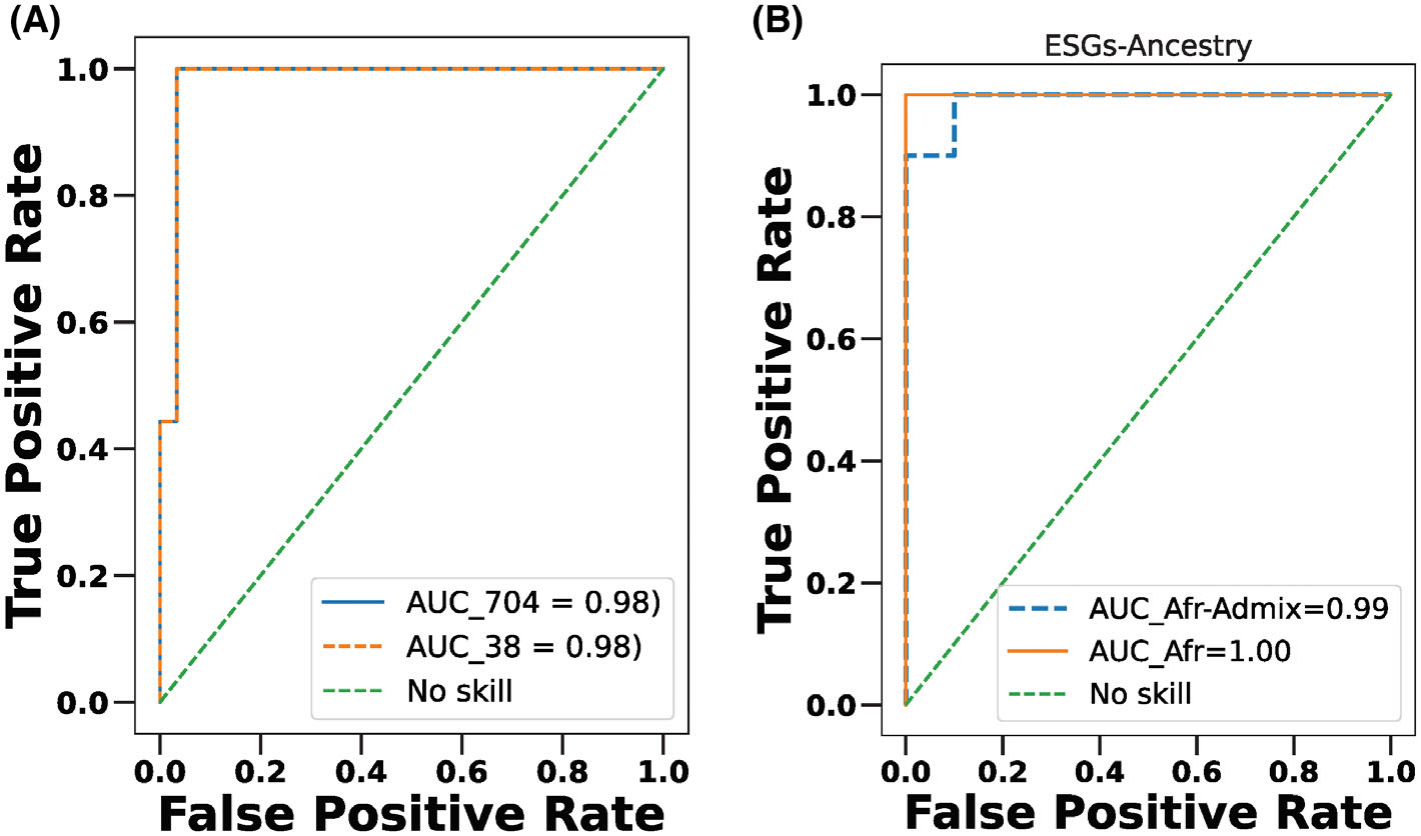
(A) Receiver Operator Characteristic curve analysis to differentiate Black endometrial tumor samples (*n* = 99) from White endometrial tumor samples (*n* = 294) using epigenetic signature genes (ESGs). (B) Receiver Operator Characteristic curve analysis using epigenetic signature genes (ESGs) after incorporating ancestry. AUC was calculated separately for African versus European (*n* = 65 in each group) and African‐Admix versus European models (*n* = 33 in each group).

With these ESGs, an AUC of 98%, sensitivity of 100% and specificity of 97% were achieved. Higher accuracy and sensitivity of the model suggest that these ESGs are main drivers of CpGs variance and can serve as predictors of differentiating TBP samples from TWP samples (Table 2, Fig. 4A).

**Table 2:** Epigenetic Signature Genes to separate endometrial tumor samples based on race and ancestry.

FilteredCpGs	Chr	Pos	Relation_to_Island	UCSC_RefGene_Name	UCSC_RefGene_Group	Regulatory_Feature_Group	PubmedID	Role
cg19933311	chrX	111158281	OpenSea	TRPC5	Body	NA	32575381	Chemoresistance
cg05084668	chr3	125655381	OpenSea	ALG1L	5′UTR	Unclassified_Cell_type_specific	35345518; 32218690	Cancer invasion
cg09651654	chr12	7781431	S_Shore	APOBEC1		NA	25085003	Cancer promotion
cg08477332	chr1	153590243	OpenSea	S100A14	TSS1500	Unclassified	3110581; 25614000	Tumor growth
cg07234876	chr8	600039	Island	ERICH1		NA		
cg09753657	chr1	159869927	Island	CCDC19	TSS200	NA	24976536	Tumor suppressor
cg09419670	chr9	123605666	S_Shore	PSMD5	TSS1500	NonGene_Associated	29716915	Tumor proteostasis
cg27299712	chr1	6550532	Island	PLEKHG5	5′UTR	NA	31309383	Cancer biomarker
cg03150409	chr4	1892317	OpenSea	WHSC1	5′UTR	NA	34445452; 34551195	Cancer progression and invasion
cg08365609	chr19	33726655	Island			Unclassified		
cg19506849	chr10	114767609	OpenSea	TCF7L2	Body	Unclassified_Cell_type_specific	35864968	Cancer metastasis
cg13910746	chr8	144365795	Island			NA		
cg11693508	chr17	37793320	Island	STARD3	TSS200	Promoter_Associated	34572920	Cancer therapy
cg25120210	chr4	3464653	N_Shore	DOK7	TSS1500	Unclassified_Cell_type_specific	33552156	Suppress tumor proliferation
cg12286861	chr10	112698410	OpenSea	SHOC2	5′UTR	NA	31974612	Cancer prognosis
cg27309611	chr5	132113725	S_Shore	SEPT8'	TSS200	NA		
cg13449967	chr11	64684301	N_Shore	ATG2A	Body	Promoter_Associated	32264916	Cancer prognosis
cg02095309	chr8	130992575	N_Shelf			NA		
cg22798247	chr6	32807372	S_Shore	TAP2	TSS1500	Promoter_Associated	35603163	Tumor‐specific immune responses
cg12502823	chr8	145754126	Island	MGC70857	Body	Promoter_Associated		
ch.17.21699132R	chr17	21775005	OpenSea			NA		
cg13367612	chr9	131683044	OpenSea	PHYHD1	TSS200	NA	31796052	Tumor invasion
cg06777732	chr12	131118426	OpenSea			NA		
cg04222842	chr1	225965391	Island	SRP9	TSS200	Unclassified	34727289	Tumor progression
cg10765909	chr12	53715428	Island	AAAS	TSS200	Promoter_Associated		
cg23117778	chr20	1206693	Island	RAD21L1	TSS200	NA		
cg05728596	chr2	219128475	OpenSea	GPBAR1	3′UTR	NA	34904217	Cancer Proliferation
cg24699670	chr19	55660614	OpenSea	TNNT1	TSS200	NA	30031058	Cancer Proliferation
cg13401893	chr6	30039432	Island	RNF39	Body	NA	31687280	Cancer biomarker
cg08207895	chr3	50388901	S_Shore	TUSC4	TSS1500	Promoter_Associated	25480944	Tumor suppressor
cg03961283	chr1	223566761	Island	C1orf65	5′UTR	Unclassified_Cell_type_specific		
cg05482050	chr5	177409334	N_Shelf			Unclassified_Cell_type_specific		
cg01513157	chr16	82031434	OpenSea	SDR42E1	3′UTR	NA	34922619	Cancer biomarker
cg00669623	chr1	1655867	Island	CDK11B	TSS200	Promoter_Associated	27049727	Cancer therapy
cg11480019	chr10	75936982	S_Shore	ADK	Body	Promoter_Associated	30944910	Cancer biomarker
cg14228592	chr8	145639181	Island	SLC39A4	Body	Promoter_Associated	32494154	Cancer biomarker
cg22681709	chr2	178499509	OpenSea	PDE11A	Body	Unclassified_Cell_type_specific	18559625	Tumor suppressor
cg04287289	chr16	89883240	Island	FANCA	TSS200	Promoter_Associated		

To test the ESGs in their accuracy to distinguish between the two racial groups using ancestry, we divided the samples into two groups. One group consisted of tumors with predominantly African ancestry, while the other included tumors with a mix of African ancestry and European ancestry (African‐admix). Importantly, these samples were not categorized based on disease aggressiveness (aggressive vs. less aggressive). Using regularized logistic regression models, we compared these groups. Interestingly, the markers consistently performed well across all tumors in separating whether they had predominantly African ancestry or a mix of African and European ancestry (Fig. 4B).

### 3.6 T‐distributed stochastic neighbor embedding (t‐SNE) analysis

**Fig. 5:**
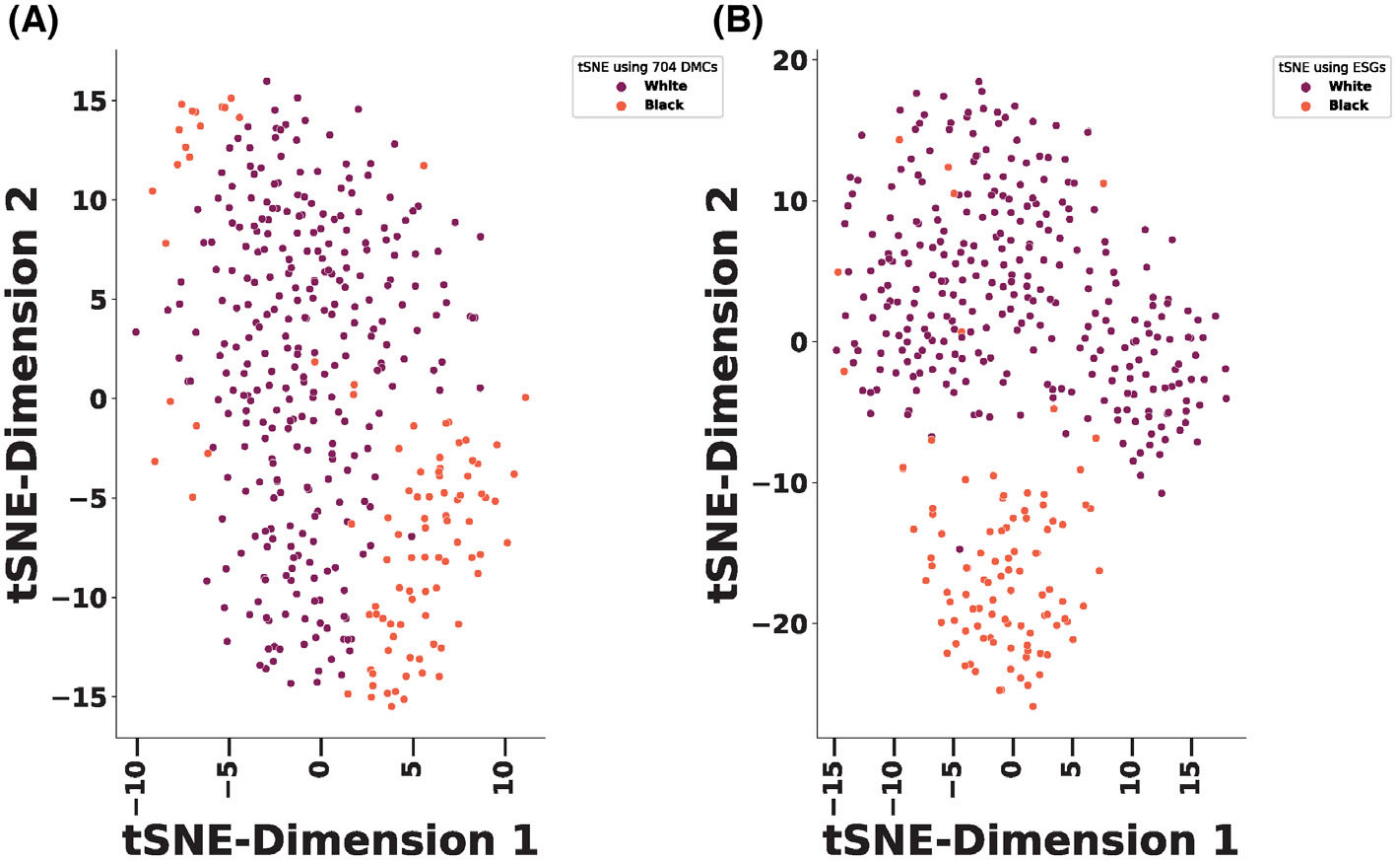
Race‐specific biomarkers classification potential revealed by t‐Distributed Stochastic Neighbor Embedding (t‐SNE). (A) The full 704 DMCs matrix embedded using t‐SNE. (B) t‐SNE Embeddings of epigenetic signature genes (ESGs). Each point represents a tumor sample. TWP samples (294) are shown in purple color and TBP samples (99) are in orange color. A total of 393 tumor samples are plotted.

To support visual exploration potential of these ESGs, we performed t‐Distributed Stochastic Neighbor Embedding (t‐SNE) analysis. We began by using all 704 DMCs. The initial t‐SNE analysis with all 704 DMCs divided the TBP and TWP into two clusters (Fig. 5A). After the initial 704 DMCs t‐SNE analysis, we used ESGs. The overall pattern of tumor samples separation with ESGs t‐SNE analysis produced a very similar view of the data further confirming that these ESGs were performing well in subsetting important structure in the data (Fig. 5B). We performed t‐SNE using this ancestry information and our results showed no difference when compared with the t‐SNE plots obtained using self‐reported race (Fig. S4).

### 3.7 Unsupervised hierarchical clustering of tumor samples based on signature genes

Unsupervised clustering of tumor samples based on signature genes showed near‐perfect separation between tumors. Most tumors with European ancestry were clustered together in cluster 1, while tumors with African and African‐admix ancestry were grouped in cluster 2, except for one African‐admix sample which clustered uniquely in cluster 3. This sample had an aggressive disease type (serous cystadenocarcinoma stage 4) and had 52% African and 48% European ancestry (Fig. S5). Clustering based on self‐reported race showed a similar pattern. The fine separation of samples into their respective groups further confirms the potential of these genes as markers associated with race and ancestry in endometrial tumor samples.

### 3.8 Performance of epigenetic signature genes in a disease‐stratified model by race and ancestry

**Fig. 6:**
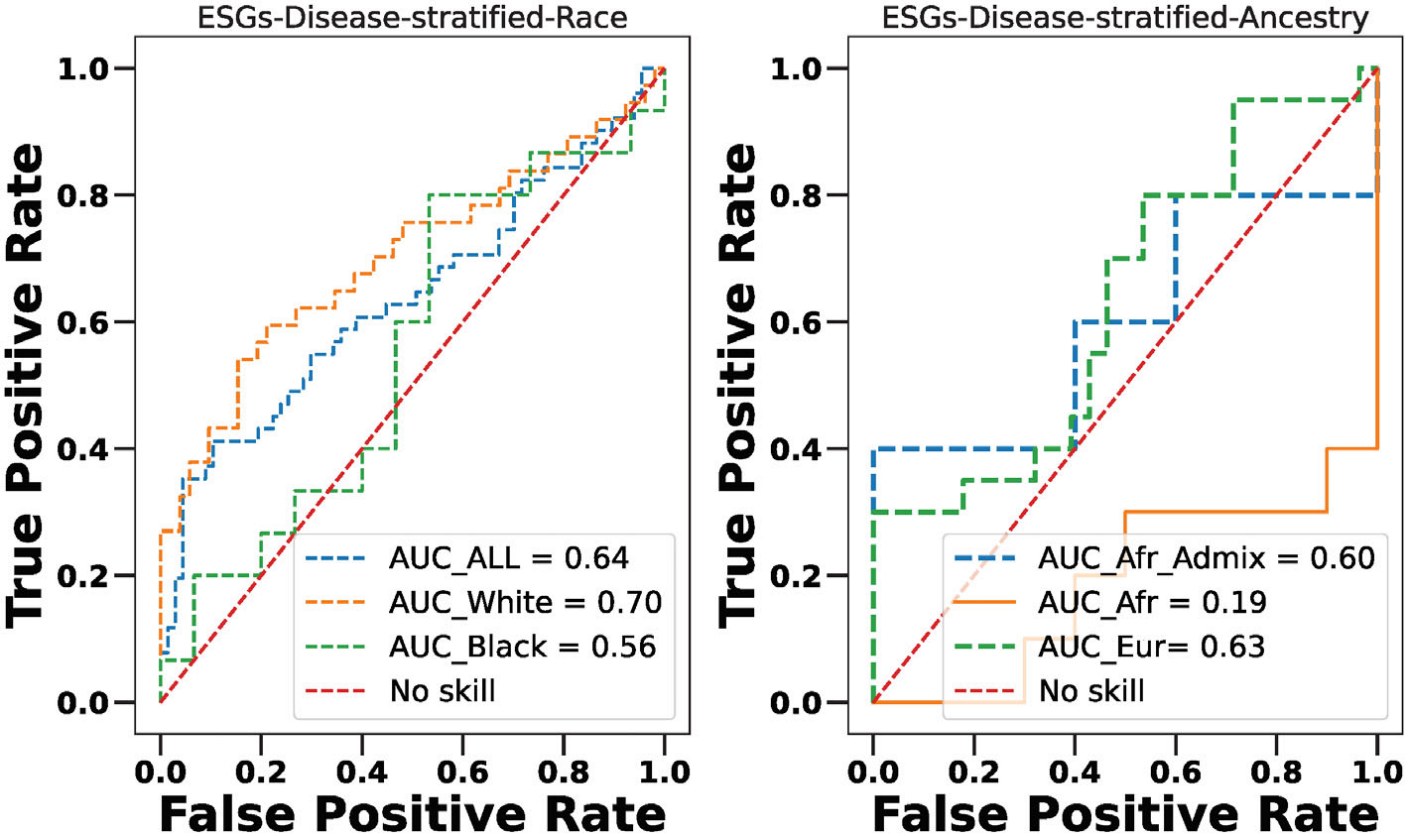
(A) Receiver Operator Characteristic curve analysis to differentiate aggressive endometrial tumor samples (*n* = 171) from less aggressive endometrial tumor samples (*n* = 222) using epigenetic signature genes (ESGs) based on race (B) Ancestry‐specific Receiver Operator Characteristic curve analysis to differentiate aggressive endometrial tumor samples from less aggressive endometrial tumor samples using epigenetic signature genes (ESGs) and ancestry. Black samples were split into two groups based on ancestral proportion (1) African (TBP with greater than 80% African ancestral proportion; *n* = 65 (aggressive = 34; less‐aggressive = 31)), (2) African‐Admix (TBP with less than 80%) African ancestral proportion (*n* = 33 (aggressive = 15; less‐aggressive = 18)) and White samples with European ancestry (*n* = 286 (aggressive = 118; less‐aggressive = 168)).

To predict the performance of ESGs based on disease aggressiveness, tumor samples were stratified into aggressive and less aggressive subtypes (see the Section 2 for details of subtype stratification), then regressed using a regularized logistic regression model. AUC based on this disease‐stratified model was 0.70 for TWP samples while in case of TBP samples the AUC dropped to 0.56. The model achieved an AUC of 0.64 when more aggressive and less aggressive samples were analyzed independent of race (Fig. 6A). These results suggest that performance of these markers according to the tumor aggressiveness is better in TWP samples than the TBP samples.

Next, we predicted the performance of ESGs based on disease aggressiveness using different proportions of African ancestry. In this disease‐stratified model, the performance of ESGs decreased as the percentage of African ancestry increased (Fig. 6B). Model that consisted mainly of African ancestry had poor performance, compared to the performance observed in European and African‐Admix (Fig. 6B).

### 3.9 K‐means clustering based redundancy filtering to find Core‐ESGs with prognostic potential

**Fig. 7:**
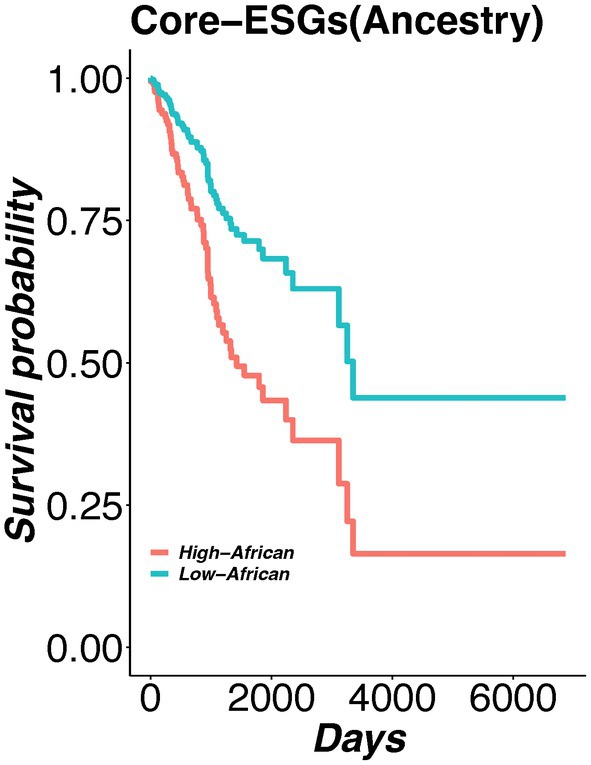
Multivariate cox regression model of survival associated core‐ESGs (APOBEC1 and PLEKHG5). Tumors with a high proportion of African ancestry (THA; *n* = 65) exhibited worse survival rates compared to those with a low proportion of African ancestry (TLA; *n* = 319).

In a subsequent step, to reduce redundancy between neighboring ESGs with similar methylation patterns in t‐SNE plots, we performed unsupervised K‐means clustering to further refine and minimize the number of ESGs. We identified three separate clusters. For filtering, we selected CpGs closest to the cluster centroid and with highest odd ratios based on logistic regression, resulting in four potential core epigenetic signature genes (Core‐ESGs) (cg19933311: TRPC5; cg09651654: APOBEC1; cg27299712: PLEKHG5; cg03150409: WHSC1). To examine the relationship between Core‐ESGs and prognosis, we conducted an overall survival analysis using both univariate and multivariate Cox proportional hazards regression models [35]. The multivariate analysis included ancestry proportions and methylation values of Core‐ESGs. Kaplan–Meier analysis showed that decreased methylation levels of two Core‐ESGs i.e., APOBEC1 and PLEKHG5 showed statistically significant overall survival differences between tumors from Black and White women, considering both race and ancestry (Likelihood ratio test; *P* value = 0.006) (Fig. 7 and Fig. S6).

### 3.10 Relationship between methylation and admixture proportion

We performed a Pearson correlation analysis using the methylation beta values of ESGs and the proportions of African and European ancestry. Interestingly, the results showed that core‐ESGs had a statistically significant negative correlation with African ancestry proportions, indicating lower methylation levels with higher African ancestry. Conversely, the methylation beta values of core‐ESGs increased with higher European ancestry proportions (Fig. S7 and Table S6).

### 3.11 Identification of potential drug targets within the ESG list

We identified nine ESG genes reported in DGIdb as potential drug targets: TNFRSF25, TRPC5, ADK, FANCA, PSMD5, GPBAR1, CDK11B, TCF7L2, and NSD2. Among these, three genes (ADK, PSMD5, and NSD2) are approved as antineoplastic agents.

## 4 Discussion

Epigenetic modification, particularly abnormal DNA methylation, which serves as a biomarker for variation in gene regulation, has great potential to transform the diagnosis and treatment of different diseases, including cancer [14]. Several studies have shown that DNA methylation differences exist between major ethnic groups, highlighting the potential contribution of epigenetic modifications to human phenotypic variation, such as response to drugs and vulnerability to common diseases [14, 36, 37].

Considering the role of DNA methylation in cancer and its potential applications in diagnosis and therapy, our study combined machine learning and differential methylation analysis to identify epigenetic signature genes (ESGs) and core‐ESGs with substantial discriminatory power between Black and White women, incorporating genetic ancestry estimations. Overall, we observed 99% European ancestry in TWP, while TBP showed an admixture of African and European ancestry, highlighting the underlying genetic heterogeneity in TBP. Women with African ancestry had a higher proportion of aggressive, advanced‐stage samples than those with African‐admixed or European ancestry. However, this difference did not reach statistical significance, likely due to the small number of self‐reported Black patients in TCGA. Further stratification of self‐reported Black into African and African‐admixed subgroups, along with cancer stage division, further reduced statistical power. Our study is in accordance with a previous report showing that African ancestry is associated with aggressive tumors (both serous and high copy number), although that study was based on demographic, genomic, and clinical data [38].

To determine ancestry‐specific methylation differences in these tumor samples, we classified them into high and low African ancestry groups. Our result showed a higher percentage of genes with hypomethylation in tumors with high African ancestry compared to in tumors with low African ancestry (69% vs. 31%). Genomic DNA hypomethylation has been recognized as a hallmark of cancer and a contributing factor in the generation of chromosomal instability by initiating heterochromatic‐euchromatic interactions, which favor oncogenic transformation [39]. This hypomethylation may also contribute to the aggressive disease type observed in tumors with high African ancestry. Our comparison with our previous self‐reported race‐based analysis focusing on methylation differences between these samples showed that 33% of these DMCs are unique to this ancestry‐specific analysis.

Pathway enrichment analysis showed that the hypomethylated genes in tumors with high African ancestry are mostly related to drug metabolism and chemical carcinogenesis‐DNA adducts. Our analysis is in agreement with previous studies, indicating that genetic diversity in drug‐metabolizing enzymes in the African population may contribute to drug‐induced adverse events and drug resistance reported in African populations compared to European populations [40, 41].

We examined the relationship between promoter hypermethylation and gene transcription using genes associated with the promoter. We discovered that 22 CpG‐gene pairs exhibited a statistically significant correlation between methylation and expression. Notably, out of these 22 pairs, 11 were unique to the ancestry‐specific analysis, differing from our previous self‐reported race‐based analysis including the ancestry‐specific CpGs (cg13648501) located in NR3C1 gene. Previous studies have shown that increased methylation at specific sites of the NR3C1 gene may contribute to vulnerability to anxiety and other psychopathologies and may moderate the effects of interventions on alcohol abuse development among African American adolescents compared with Europeans [42, 43, 44, 45].

To shortlist potential methylation signature genes, we screened all DMCs by integrating race and ancestry using machine learning techniques. We used recursive feature selection to filter candidate epigenetic signature genes (ESGs). To evaluate the performance of ESGs in separating tumor samples based on DNA methylation, we developed a comprehensive model (without adjusting for disease phenotype) and a stratified model. In our comprehensive model, the ESGs performance was strongly correlated with self‐reported race and ancestry. However, in our disease‐stratified model, ESGs showed improved performance in tumor samples with decrease African ancestry proportion, that is, European, and African‐admixed ancestry. One potential explanation for the relatively diminished performance of our proposed ESGs in patients with African ancestry is the increased underlying genetic diversity and reduced linkage disequilibrium [46, 47], which likely contribute to greater methylation variability in Black aggressive tumor samples compared to White aggressive tumor samples, as highlighted in our previous analysis [20]. We then performed redundancy filtering using unsupervised KMeans clustering to further filter the Core‐ESGs. Survival analysis based on methylation values of Core‐ESGs, that is, APOBEC1 and PLEKHG5 showed that tumors with high African ancestry had poorer survival than tumors with low African ancestry, which is in agreement with previous studies on survival, where patients with increased levels of African ancestry showed worse outcomes [4, 48]. Both PLEKHG5 and APOBEC1 were previously suggested as valuable prognostic biomarkers and potential anti‐tumor targets for different cancers [46, 49]. PLEKHG5 (Pleckstrin homology and RhoGEF domain containing G5) gene has been reported to promote tumor cell migration and invasion [47] while APOBEC1 (Apolipoprotein B mRNA editing enzyme, catalytic polypeptide 1) was shown to exert its oncogenic potential by triggering genetic alterations as reported by a study on esophageal adenocarcinomas [46].

### 4.1 Strengths and limitation

The major strengths of this study lie in integrating both race and ancestry proportions of tumor samples with machine learning techniques and leveraging large datasets like TCGA to identify biomarkers for targeted endometrial cancer treatments, whereas previous studies have solely relied on race. Unlike previous studies that have compared tumor vs. normal samples, this study focuses exclusively on tumor samples to explore tumor‐specific changes in these two ancestral/racial groups. Although an in‐depth *in silico* analysis was performed to shortlist ESGs and Core‐ESGs, the study is limited by a small number of tumors from Black women, which was further reduced when samples were categorized into high and low African ancestry proportions. Additionally, we have not yet located another large dataset to further validate the performance of these ESGs. Future research should focus on validating these markers in larger sample size, particularly focusing on Black tumor samples, to fully understand their clinical usefulness and to develop more effective, ancestry‐specific diagnostic tools.

## 5 Conclusion

Expanding on our previous research into methylation markers, we employed machine learning techniques to integrate ancestry into the differential methylation analysis of TCGA data. By combining differential methylation analysis and machine learning techniques, we were able to shortlist highly robust and selective DMCs (referred to here as ESGs and Core‐ESGs) in Black and White women with endometrial cancer. Our findings indicate that these ESGs can efficiently differentiate between aggressive and less aggressive tumors within the TWP, considering both self‐reported race and ancestry. However, their performance may be more limited in TBP, particularly when integrating genetic ancestral proportions. An increase in sample size for TBP would be required. We confirmed our previous finding of increased methylation hypervariability in Black women using ESGs in the disease‐stratified model. Methylation levels of ESGs showed significant associations with survival outcomes, with THA exhibiting decreased methylation linked to worse overall survival (OS) compared to TLA. We proposed that these novel signature genes can be used as potential therapeutic targets for diagnosis and treating Black and White women with endometrial cancer and can serve as novel race/ancestry biomarker for determining the prognosis of patients with endometrial cancer.

## Conflict of interest

The authors declare no conflict of interest.

## Author contributions

HA: conceptualization, methodology, formal analysis, visualization, writing – original draft. JJK: supervision, methodology, formal analysis, conceptualization, review, and editing.

## Supporting information


**Fig. S1.** Venn diagram showing shared and unique DMCs from race and ancestry analyses.
**Fig. S2.** (A) KEGG pathway enrichment analysis of hypomethylated genes in tumors from patients of European ancestry. (B) Epigenetic Heterogeneity in African‐Admixed Populations. (Left) PCA analysis reveals that African‐admixed samples display diverse methylation profiles, with some clustering closer to European samples (blue), others resembling African samples (red), and some forming distinct clusters (e.g., PC1 ≥ 0.20 & PC1 ≤ 0.33 & PC2 < 0). (Right): Comparison of methylation beta values between African and African‐admixed individuals.
**Fig. S3.** Gene Set Enrichment Analysis of Differentially Expressed Genes. Pathways with a *P* value < 5% and FDR *q*‐value ≤ 25 are shown.
**Fig. S4.** (A) Comparison of t‐SNE Embeddings of epigenetic signature genes (ESGs) using self‐reported race and ancestry (First model: European vs. African). Each point represents a tumor sample. White tumor samples (And European ancestry: 64 tumor samples) are shown in orange color and Black tumor samples (African ancestry: 64 tumor sample with > 80% African ancestry proportion) are in purple color. A total of 128 tumor samples are plotted. (B) Comparison of t‐SNE Embeddings of epigenetic signature genes (ESGs) using self‐reported race and ancestry (Second model: European vs. African‐Admix). Each point represents a tumor sample. White tumor samples (and European ancestry: 32 tumor samples) are shown in orange color and Black tumor samples (African ancestry: 32 tumor sample with < 80% African proportion) are in purple color. A total of 64 tumor samples are plotted.
**Fig. S5.** Unsupervised hierarchical clustering of endometrial tumor samples based on methylation beta values of ESGs. Black endometrial tumor samples (Purple) and White endometrial tumor samples (Green) are in rows. The dendrogram shows the Euclidean distance between individual tumor samples.
**Fig. S6.** Multivariate cox regression model of survival associated core‐ESGs based on race (APOBEC1 and PLEKHG5). TBP exhibited worse survival rates compared to TWP.
**Fig. S7.** (A) Correlation of ESGs methylation with African ancestry. (B) Correlation of ESGs methylation with European ancestry. Correlations are color‐coded, with more significant ones in blue and significant ones in red. Red boxes represent four potential core epigenetic signature genes (Core‐ESGs) (cg19933311: TRPC5; cg09651654: APOBEC1; cg27299712: PLEKHG5; cg03150409: WHSC1).

**Table S1.** Differentially methylated CPGs (DMCs) genes (Tumors from Low Africans (TLA) vs. Tumors from High Africans (THA)).
**Table S2.** Common DMCs based on race and ancestry analysis.
**Table S3.** Differentially expressed genes (DEGs) (Tumors from Low Africans (TLA) vs. Tumors from High Africans (THA)).
**Table S4.** (A) CpGs‐Gene pairs with absolute mean methylation differences (delta) greater than 10% within the promoter region. (B) Ancestry‐specific CpGs‐Gene pairs with absolute mean methylation differences (delta) greater than 10% within the promoter region.
**Table S5.** Race‐specific Differentially methylated CPGs (DMCs) genes (Tumors from white vs. Tumors from black).
**Table S6.** (A) Correlation of core‐ESGs methylation with African ancestry. (B) Correlation of core‐ESGs methylation with European ancestry.

## Data Availability

Data from The Cancer Genome Atlas (TCGA) were acquired from dbGap following written approval (Study Accession: phs000178.v11.p8.c1). The datasets generated and analyzed during the current study are included within the article and its additional files.

## References

[1] Zhang B , Shi H , Wang H . Machine learning and AI in cancer prognosis, prediction, and treatment selection: a critical approach. J Multidiscip Healthc. 2023;16:1779–1791. 37398894 10.2147/JMDH.S410301 PMC10312208

[2] Hart GR , Yan V , Huang GS , Liang Y , Nartowt BJ , Muhammad W , et al. Population‐based screening for endometrial cancer: human vs. machine intelligence. Front Artif Intell. 2020;3:539879. 33733200 10.3389/frai.2020.539879 PMC7861326

[3] Siegel RL , Giaquinto AN , Jemal A . Cancer statistics, 2024. CA Cancer J Clin. 2024;74:12–49. 38230766 10.3322/caac.21820

[4] Pinheiro PS , Medina HN , Koru‐Sengul T , Qiao B , Schymura M , Kobetz EN , et al. Endometrial cancer type 2 incidence and survival disparities within subsets of the US black population. Front Oncol. 2021;11:699577. 34354948 10.3389/fonc.2021.699577 PMC8329656

[5] Berek JS , Matias‐Guiu X , Creutzberg C , Fotopoulou C , Gaffney D , Kehoe S , et al. FIGO staging of endometrial cancer: 2023. Int J Gynaecol Obstet. 2023;162(2):383–394. 37337978 10.1002/ijgo.14923

[6] Cote ML , Ruterbusch JJ , Olson SH , Lu K , Ali‐Fehmi R . The growing burden of endometrial cancer: a major racial disparity affecting black women. Cancer Epidemiol Biomarkers Prev. 2015;24(9):1407–1415. 26290568 10.1158/1055-9965.EPI-15-0316

[7] Farley J , Risinger JI , Rose GS , Maxwell GL . Racial disparities in blacks with gynecologic cancers. Cancer. 2007;110(2):234–243. 17559136 10.1002/cncr.22797

[8] Park AB , Darcy KM , Tian C , Casablanca Y , Schinkel JK , Enewold L , et al. Racial disparities in survival among women with endometrial cancer in an equal access system. Gynecol Oncol. 2021;163(1):125–129. 34325938 10.1016/j.ygyno.2021.07.022 PMC8562590

[9] Mukerji B , Baptiste C , Chen L , Tergas AI , Hou JY , Ananth CV , et al. Racial disparities in young women with endometrial cancer. Gynecol Oncol. 2018;148(3):527–534. 29307452 10.1016/j.ygyno.2017.12.032 PMC5829000

[10] Dubil EA , Tian C , Wang G , Tarney CM , Bateman NW , Levine DA , et al. Racial disparities in molecular subtypes of endometrial cancer. Gynecol Oncol. 2018;149(1):106–116. 29605044 10.1016/j.ygyno.2017.12.009

[11] Guttery DS , Blighe K , Polymeros K , Symonds RP , Macip S , Moss EL . Racial differences in endometrial cancer molecular portraits in the cancer genome atlas. Oncotarget. 2018;9(24):17093–17103. 29682207 10.18632/oncotarget.24907 PMC5908308

[12] Chan MH , Merrill SM , Konwar C , Kobor MS . An integrative framework and recommendations for the study of DNA methylation in the context of race and ethnicity. Discov Soc Sci Health. 2023;3(1):9. 37122633 10.1007/s44155-023-00039-z PMC10118232

[13] Rosenberg NA , Pritchard JK , Weber JL , Cann HM , Kidd KK , Zhivotovsky LA , et al. Genetic structure of human populations. Science. 2002;298(5602):2381–2385. 12493913 10.1126/science.1078311

[14] Schübeler D . Function and information content of DNA methylation. Nature. 2015;517(7534):321–326. 25592537 10.1038/nature14192

[15] Fagny M , Patin E , MacIsaac JL , Rotival M , Flutre T , Jones MJ , et al. The epigenomic landscape of African rainforest hunter‐gatherers and farmers. Nat Commun. 2015;6:10047. 26616214 10.1038/ncomms10047 PMC4674682

[16] Ehrlich M . DNA hypomethylation in cancer cells. Epigenomics. 2009;1(2):239–259. 20495664 10.2217/epi.09.33 PMC2873040

[17] Su J , Huang YH , Cui X , Wang X , Zhang X , Lei Y , et al. Homeobox oncogene activation by pan‐cancer DNA hypermethylation. Genome Biol. 2018;19(1):108. 30097071 10.1186/s13059-018-1492-3 PMC6085761

[18] Costello JF , Frühwald MC , Smiraglia DJ , Rush LJ , Robertson GP , Gao X , et al. Aberrant CpG‐Island methylation has non‐random and tumour‐type‐specific patterns. Nat Genet. 2000;24(2):132–138. 10655057 10.1038/72785

[19] Huo X , Sun H , Cao D , Yang J , Peng P , Yu M , et al. Identification of prognosis markers for endometrial cancer by integrated analysis of DNA methylation and RNA‐Seq data. Sci Rep. 2019;9(1):9924. 31289358 10.1038/s41598-019-46195-8 PMC6617448

[20] Asif H , Foley G , Simon M , Roque D , Kim JJ . Analysis of endometrial carcinoma TCGA reveals differences in DNA methylation in tumors from black and white women. Gynecol Oncol. 2022;170:1–10. 36580834 10.1016/j.ygyno.2022.12.011 PMC10023328

[21] Tomczak K , Czerwińska P , Wiznerowicz M . The cancer genome atlas (TCGA): an immeasurable source of knowledge. Contemp Oncol (Pozn). 2015;19(1A):A68–A77. 25691825 10.5114/wo.2014.47136 PMC4322527

[22] Colaprico A , Silva TC , Olsen C , Garofano L , Cava C , Garolini D , et al. TCGAbiolinks: an R/Bioconductor package for integrative analysis of TCGA data. Nucleic Acids Res. 2016;44(8):e71. 26704973 10.1093/nar/gkv1507 PMC4856967

[23] Chen YA , Lemire M , Choufani S , Butcher DT , Grafodatskaya D , Zanke BW , et al. Discovery of cross‐reactive probes and polymorphic CpGs in the Illumina Infinium HumanMethylation450 microarray. Epigenetics. 2013;8(2):203–209. 23314698 10.4161/epi.23470 PMC3592906

[24] Du P , Zhang X , Huang C‐C , Jafari N , Kibbe WA , Hou L , et al. Comparison of Beta‐value and M‐value methods for quantifying methylation levels by microarray analysis. BMC Bioinformatics. 2010;11:587. 21118553 10.1186/1471-2105-11-587 PMC3012676

[25] Carrot‐Zhang J , Chambwe N , Damrauer JS , Knijnenburg TA , Robertson AG , Yau C , et al. Comprehensive analysis of genetic ancestry and its molecular correlates in cancer. Cancer Cell. 2020;37(5):639–654.e6. 32396860 10.1016/j.ccell.2020.04.012 PMC7328015

[26] Purcell S , Neale B , Todd‐Brown K , Thomas L , Ferreira MAR , Bender D , et al. PLINK: a tool set for whole‐genome association and population‐based linkage analyses. Am J Hum Genet. 2007;81(3):559–575. 17701901 10.1086/519795 PMC1950838

[27] Romanel A , Zhang T , Elemento O , Demichelis F . EthSEQ: ethnicity annotation from whole exome sequencing data. Bioinformatics. 2017;33(15):2402–2404. 28369222 10.1093/bioinformatics/btx165 PMC5818140

[28] Alexander DH , Novembre J , Lange K . Fast model‐based estimation of ancestry in unrelated individuals. Genome Res. 2009;19(9):1655–1664. 19648217 10.1101/gr.094052.109 PMC2752134

[29] Lek M , Karczewski KJ , Minikel EV , Samocha KE , Banks E , Fennell T , et al. Analysis of protein‐coding genetic variation in 60,706 humans. Nature. 2016;536(7616):285–291. 27535533 10.1038/nature19057 PMC5018207

[30] Law CW , Alhamdoosh M , Su S , Dong X , Tian L , Smyth GK , et al. RNA‐seq analysis is easy as 1‐2‐3 with limma, Glimma and edgeR. F1000Res. 2016;5. 10.12688/f1000research.9005.3 PMC4937821 27441086

[31] Saeys Y , Inza I , Larrañaga P . A review of feature selection techniques in bioinformatics. Bioinformatics. 2007;23(19):2507–2517. 17720704 10.1093/bioinformatics/btm344

[32] Piao Y , Piao M , Park K , Ryu KH . An ensemble correlation‐based gene selection algorithm for cancer classification with gene expression data. Bioinformatics. 2012;28(24):3306–3315. 23060613 10.1093/bioinformatics/bts602

[33] Flynn R . Survival analysis. J Clin Nurs. 2012;21(19–20):2789–2797. 22860755 10.1111/j.1365-2702.2011.04023.x

[34] Gershon ES , Lee SH , Zhou X , Sweeney JA , Tamminga C , Pearlson GA , et al. An opportunity for primary prevention research in psychotic disorders. Schizophr Res. 2021;243:433–439. 34315649 10.1016/j.schres.2021.07.001 PMC8784565

[35] Zhang MJ . Cox proportional hazards regression models for survival data in cancer research. Cancer Treat Res. 2002;113:59–70. 12613350 10.1007/978-1-4757-3571-0_4

[36] Fraser HB , Lam LL , Neumann SM , Kobor MS . Population‐specificity of human DNA methylation. Genome Biol. 2012;13(2):R8. 22322129 10.1186/gb-2012-13-2-r8 PMC3334571

[37] Heyn H , Moran S , Hernando‐Herraez I , Sayols S , Gomez A , Sandoval J , et al. DNA methylation contributes to natural human variation. Genome Res. 2013;23(9):1363–1372. 23908385 10.1101/gr.154187.112 PMC3759714

[38] Sanchez‐Covarrubias AP , Tabuyo‐Martin AD , George S , Schlumbrecht M . African ancestry is associated with aggressive endometrial cancer. Am J Obstet Gynecol. 2023;228(1):92–95.e10. 35944605 10.1016/j.ajog.2022.07.040

[39] Cappetta M , Berdasco M , Hochmann J , Bonilla C , Sans M , Hidalgo PC , et al. Effect of genetic ancestry on leukocyte global DNA methylation in cancer patients. BMC Cancer. 2015;15:434. 26012346 10.1186/s12885-015-1461-0 PMC4445803

[40] Rajman I , Knapp L , Morgan T , Masimirembwa C . African genetic diversity: implications for cytochrome P450‐mediated drug metabolism and drug development. EBioMedicine. 2017;17:67–74. 28237373 10.1016/j.ebiom.2017.02.017 PMC5360579

[41] Wright GEB , Carleton B , Hayden MR , Ross CJD . The global spectrum of protein‐coding pharmacogenomic diversity. Pharmacogenomics J. 2018;18(1):187–195. 27779249 10.1038/tpj.2016.77 PMC5817389

[42] Zheng Y , Albert D , McMahon RJ , Dodge K , Dick D , Conduct Problems Prevention Research Group . Glucocorticoid receptor (NR3C1) gene polymorphism moderate intervention effects on the developmental trajectory of African–American adolescent alcohol abuse. Prev Sci. 2018;19(1):79–89. 27817096 10.1007/s11121-016-0726-4 PMC5420337

[43] Zannas AS , Arloth J , Carrillo‐Roa T , Iurato S , Röh S , Ressler KJ , et al. Lifetime stress accelerates epigenetic aging in an urban, African American cohort: relevance of glucocorticoid signaling. Genome Biol. 2015;16:266. 26673150 10.1186/s13059-015-0828-5 PMC4699359

[44] Clarke LS , Corwin EJ , Dunlop AL , Hankus A , Bradner JM , Paul S , et al. Glucocorticoid receptor sensitivity in early pregnancy in an African American cohort. Am J Reprod Immunol. 2020;84(1):e13252. 32320110 10.1111/aji.13252 PMC7416519

[45] Wadji DL , Morina N , Martin‐Soelch C , Wicky C . Methylation of the glucocorticoid receptor gene (NR3C1) in dyads mother‐child exposed to intimate partner violence in Cameroon: association with anxiety symptoms. PLoS One. 2023;18(4):e0273602. 37023023 10.1371/journal.pone.0273602 PMC10079034

[46] Saraconi G , Severi F , Sala C , Mattiuz G , Conticello SG . The RNA editing enzyme APOBEC1 induces somatic mutations and a compatible mutational signature is present in esophageal adenocarcinomas. Genome Biol. 2014;15(7):417. 25085003 10.1186/s13059-014-0417-z PMC4144122

[47] Grun D , Adhikary G , Eckert RL . NRP‐1 interacts with GIPC1 and SYX to activate p38 MAPK signaling and cancer stem cell survival. Mol Carcinog. 2019;58(4):488–499. 30456845 10.1002/mc.22943 PMC6417965

[48] Rocconi RP , Lankes HA , Brady WE , Goodfellow PJ , Ramirez NC , Alvarez RD , et al. The role of racial genetic admixture with endometrial cancer outcomes: an NRG oncology/gynecologic oncology group study. Gynecol Oncol. 2016;140(2):264–269. 26603970 10.1016/j.ygyno.2015.11.018 PMC4842318

[49] Qian M , Chen Z , Wang S , Guo X , Zhang Z , Qiu W , et al. PLEKHG5 is a novel prognostic biomarker in glioma patients. Int J Clin Oncol. 2019;24(11):1350–1358. 31309383 10.1007/s10147-019-01503-0

