# Supplementary material for: Comparing self‐reported race and genetic ancestry for identifying potential differentially methylated sites in endometrial cancer: insights from African ancestry proportions using machine learning models
Source: Mol Oncol. 2025 Mar 6;19(12):3596–612. doi: 10.1002/1878-0261.70013 (PMC12688174; doi:10.1002/1878-0261.70013)
Supplement: Supplementary file 1 — Fig. S1. Venn diagram showing shared and unique DMCs from race and ancestry analyses. Fig. S2. (A) KEGG pathway enrichment analysis of hypomethylated genes in tumors from patients of European ancestry. (B) Epigenetic Heterogeneity in African‐Admixed Populations. (Left) PCA analysis reveals that African‐admixed samples display diverse methylation profiles, with some clustering closer to European samples (blue), others resembling African samples (red), and some forming distinct clusters (e.g., PC1 ≥ 0.20 & PC1 ≤ 0.33 & PC2 < 0). (Right): Comparison of methylation beta values between African and African‐admixed individuals. Fig. S3. Gene Set Enrichment Analysis of Differentially Expressed Genes. Pathways with a P value < 5% and FDR q‐value ≤ 25 are shown. Fig. S4. (A) Comparison of t‐SNE Embeddings of epigenetic signature genes (ESGs) using self‐reported race and ancestry (First model: European vs. African). Each point represents a tumor sample. White tumor samples (And European ancestry: 64 tumor samples) are shown in orange color and Black tumor samples (African ancestry: 64 tumor sample with > 80% African ancestry proportion) are in purple color. A total of 128 tumor samples are plotted. (B) Comparison of t‐SNE Embeddings of epigenetic signature genes (ESGs) using self‐reported race and ancestry (Second model: European vs. African‐Admix). Each point represents a tumor sample. White tumor samples (and European ancestry: 32 tumor samples) are shown in orange color and Black tumor samples (African ancestry: 32 tumor sample with < 80% African proportion) are in purple color. A total of 64 tumor samples are plotted. Fig. S5. Unsupervised hierarchical clustering of endometrial tumor samples based on methylation beta values of ESGs. Black endometrial tumor samples (Purple) and White endometrial tumor samples (Green) are in rows. The dendrogram shows the Euclidean distance between individual tumor samples. Fig. S6. Multivariate cox regression model of survival associated co [file MOL2-19-3596-s002.pdf]

Supplementary Figures

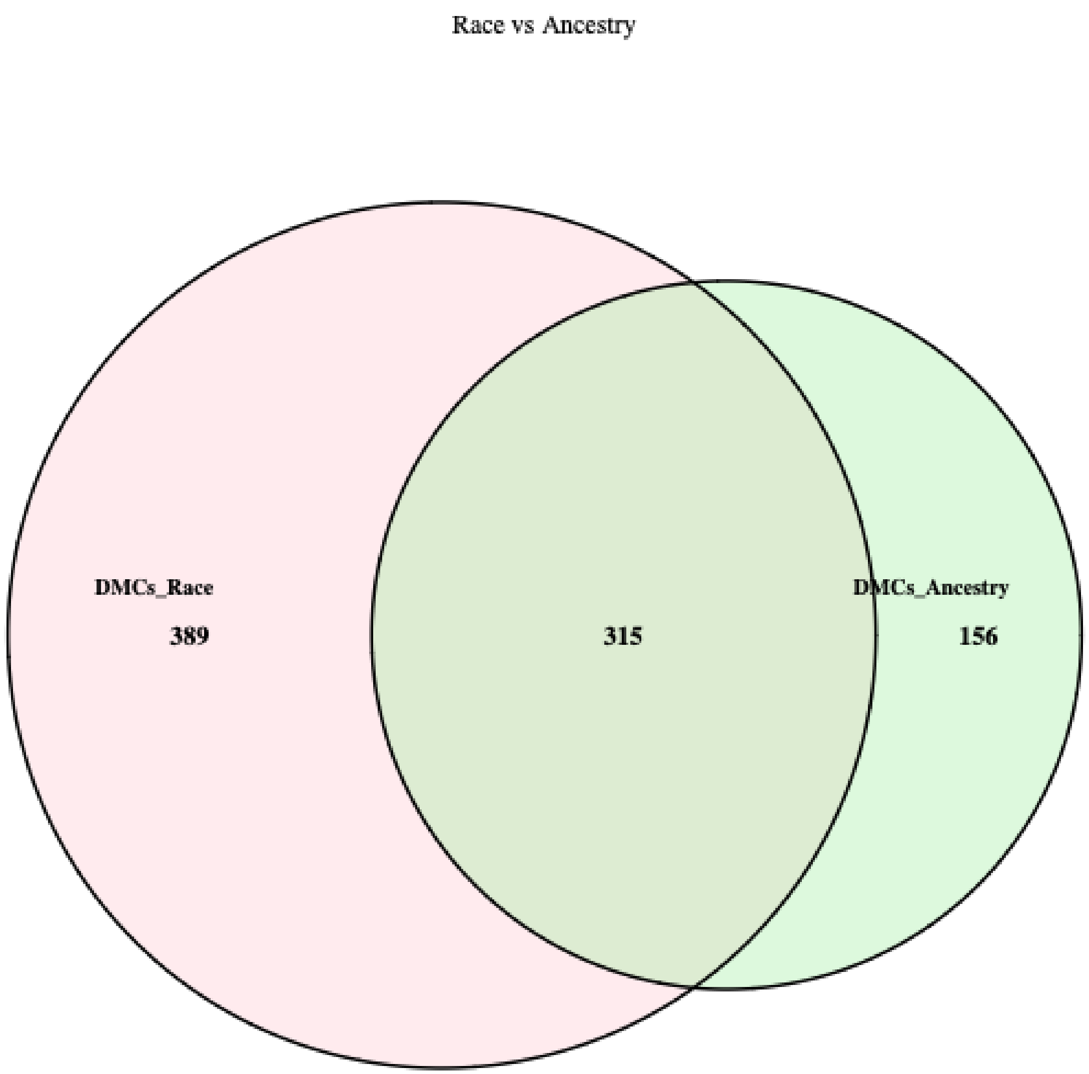

Supplementary Figure S1

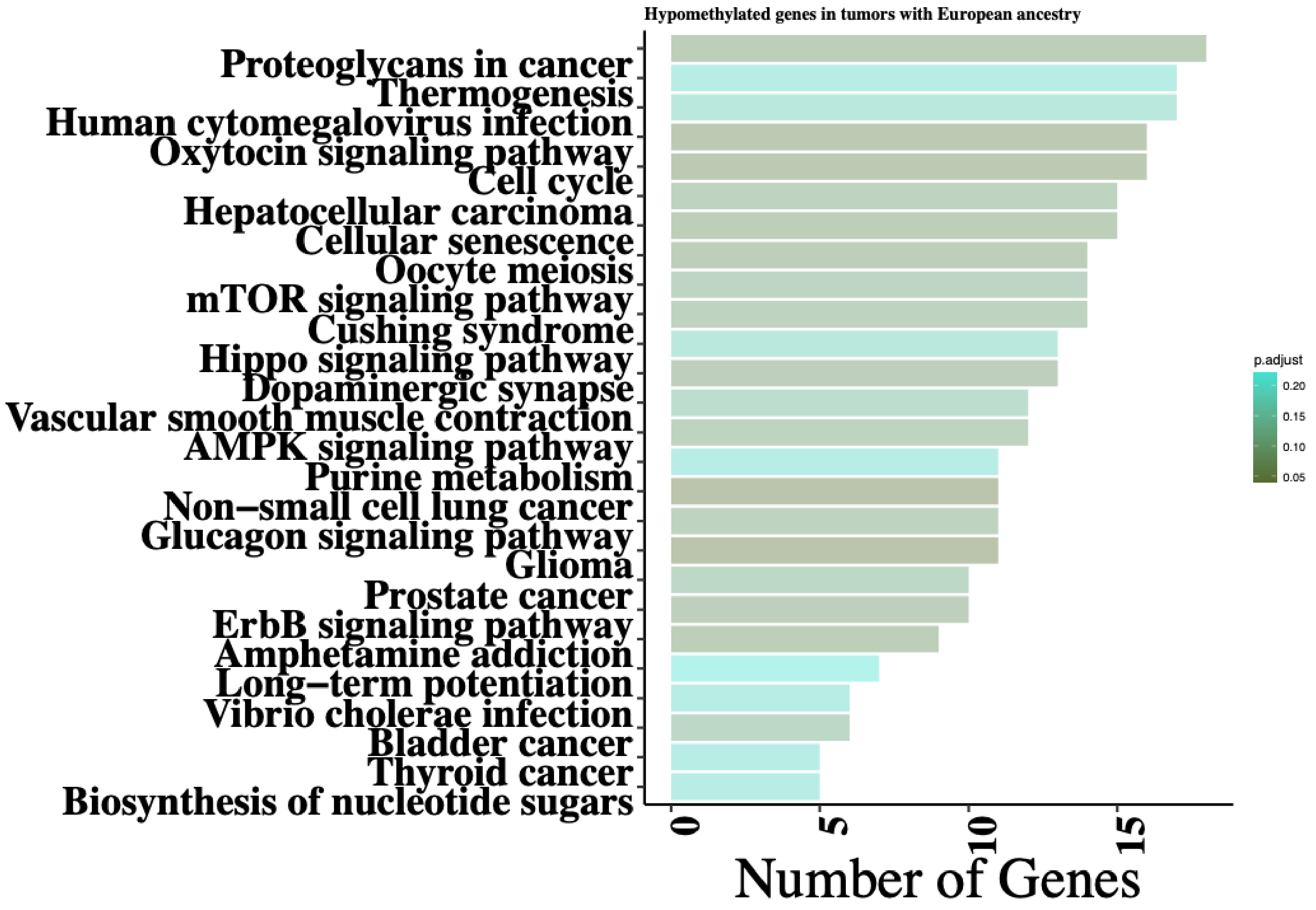

Supplementary Figure S2A

Did the authors try to analyze the diversity within the AA subset of patients?

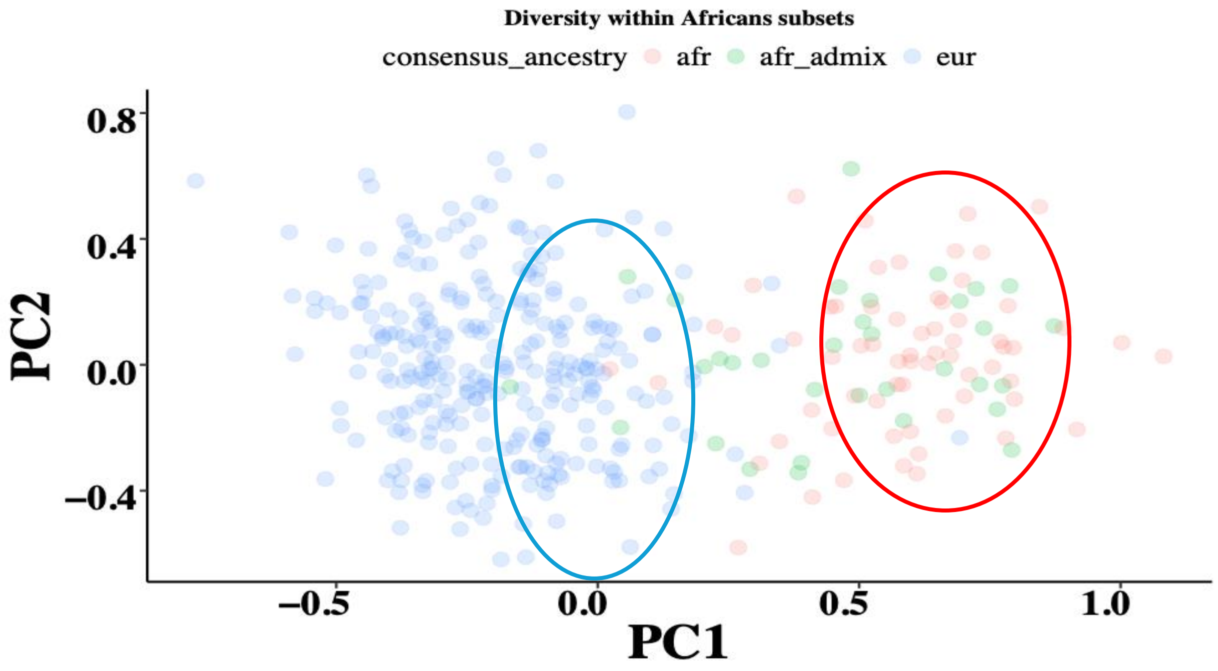

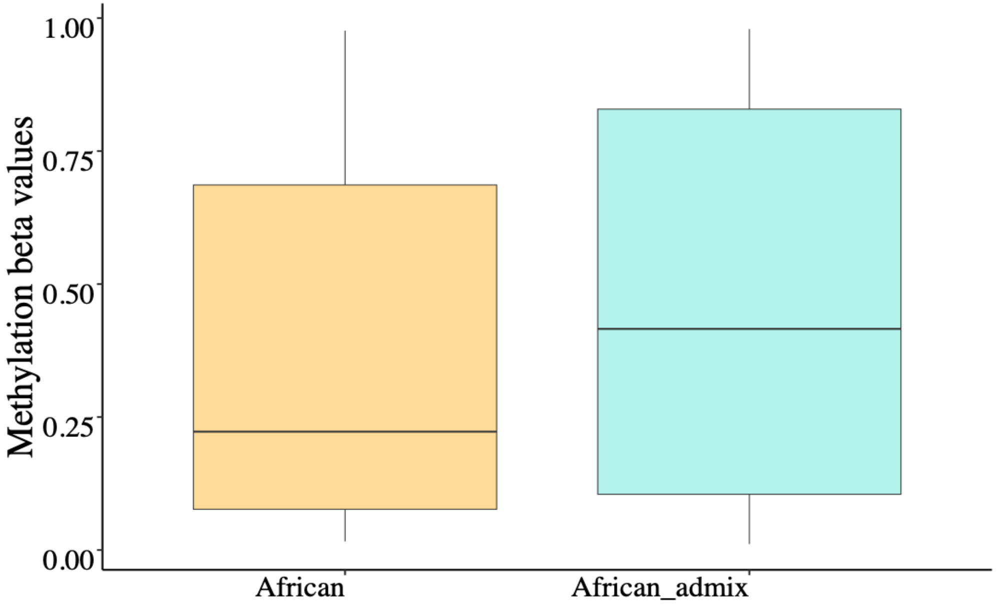

Supplementary Figure S2B

.

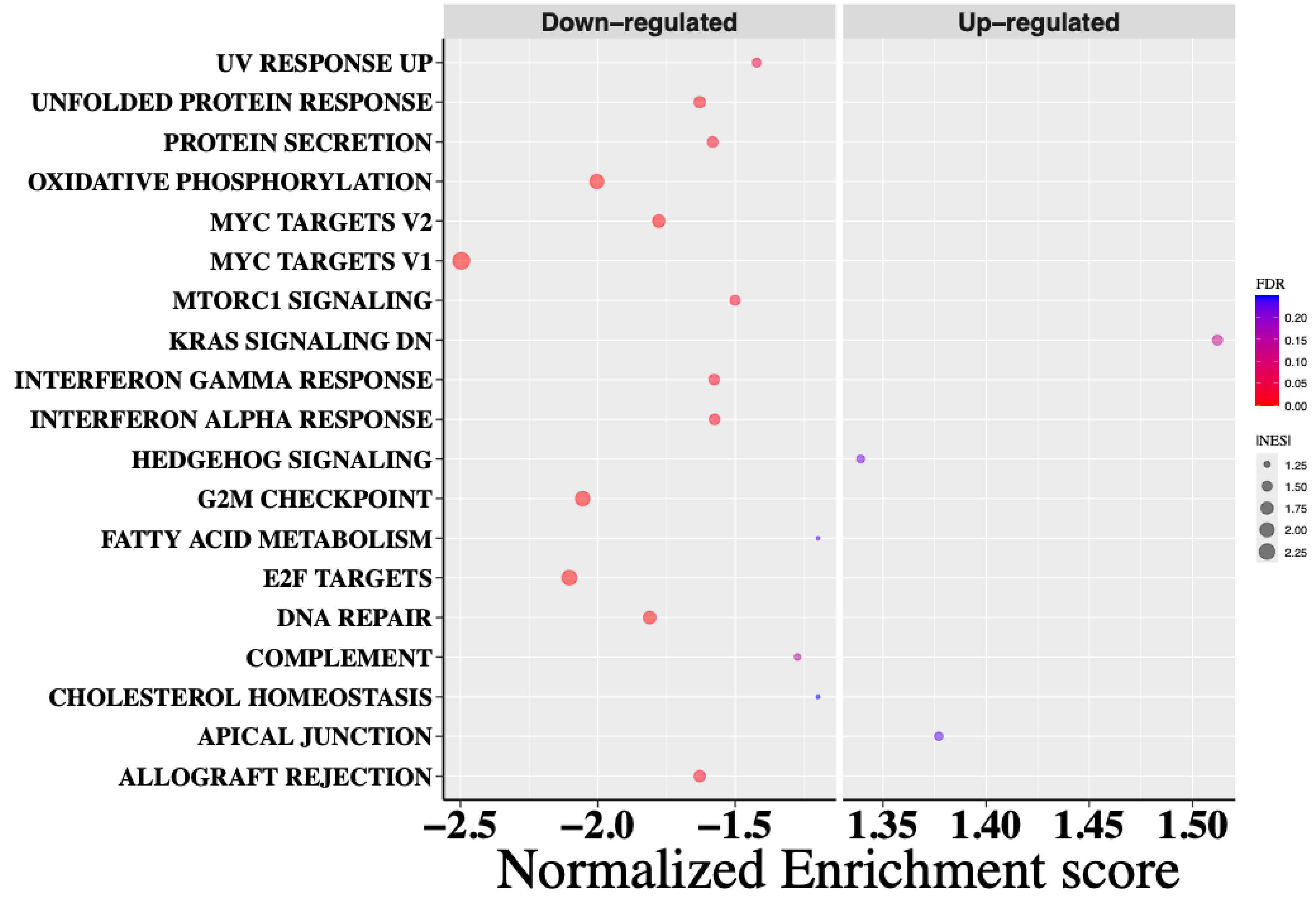

Supplementary Figure S3

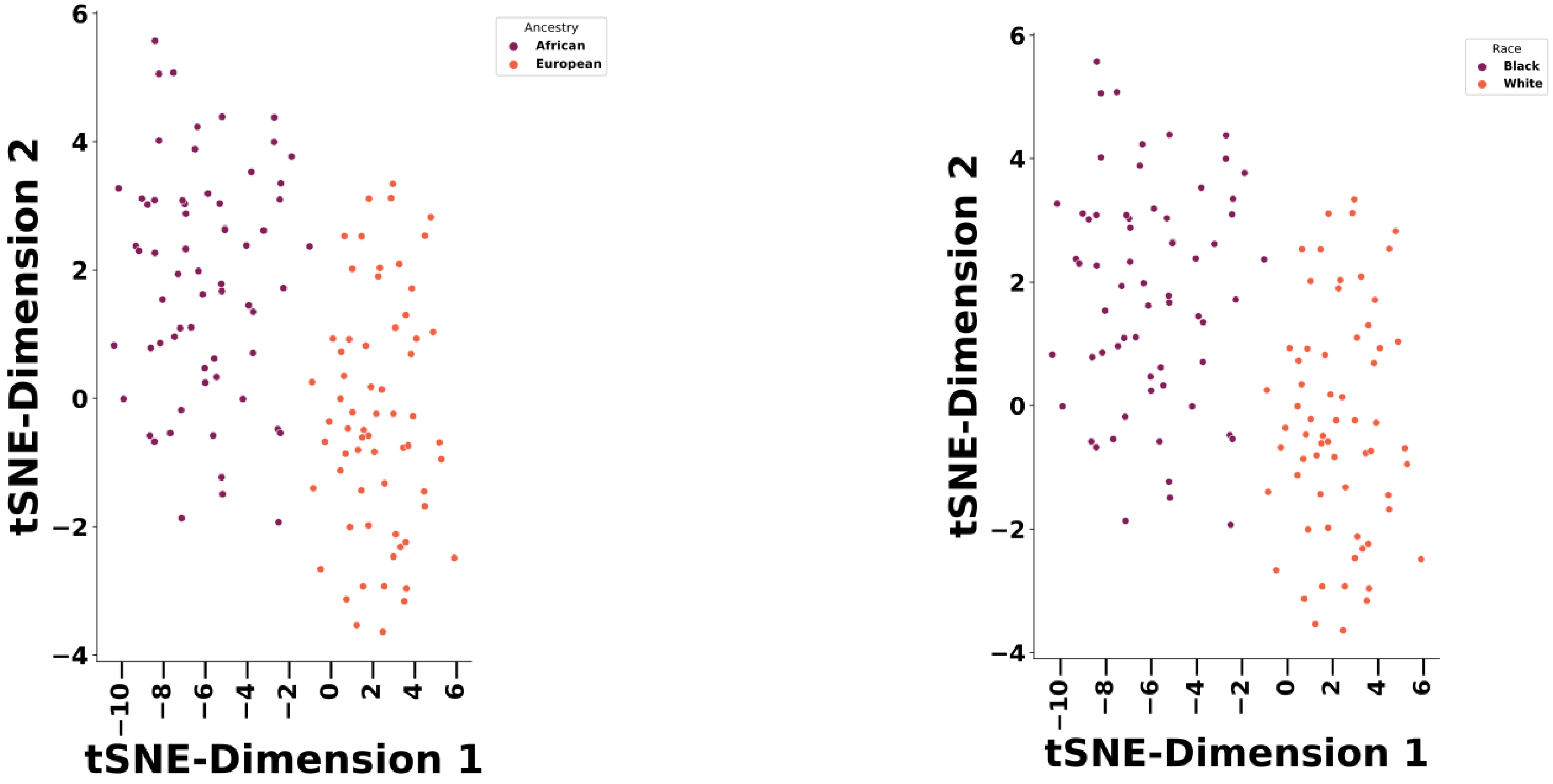

Supplementary Figure S4A

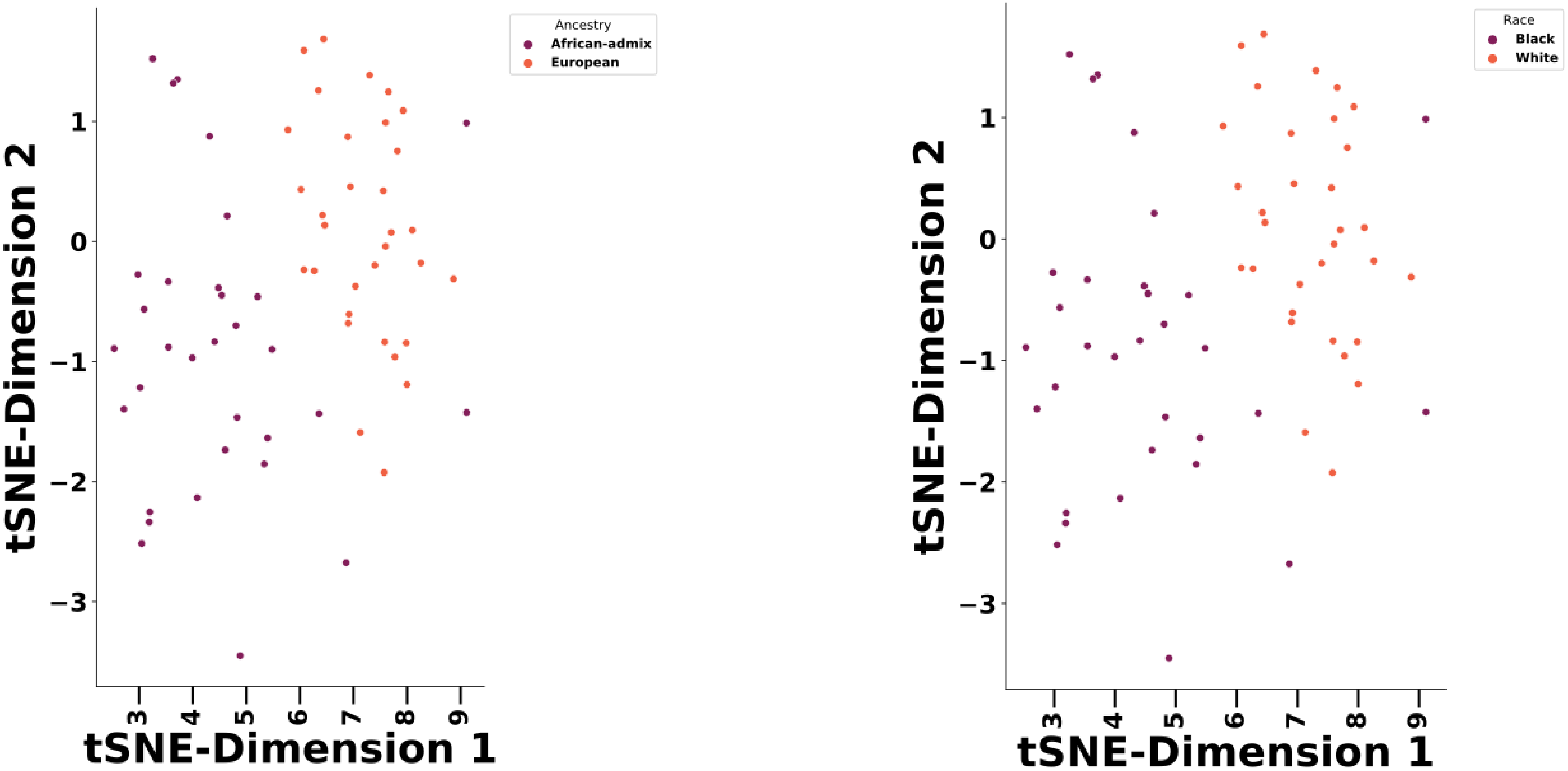

Supplementary Figure S4B

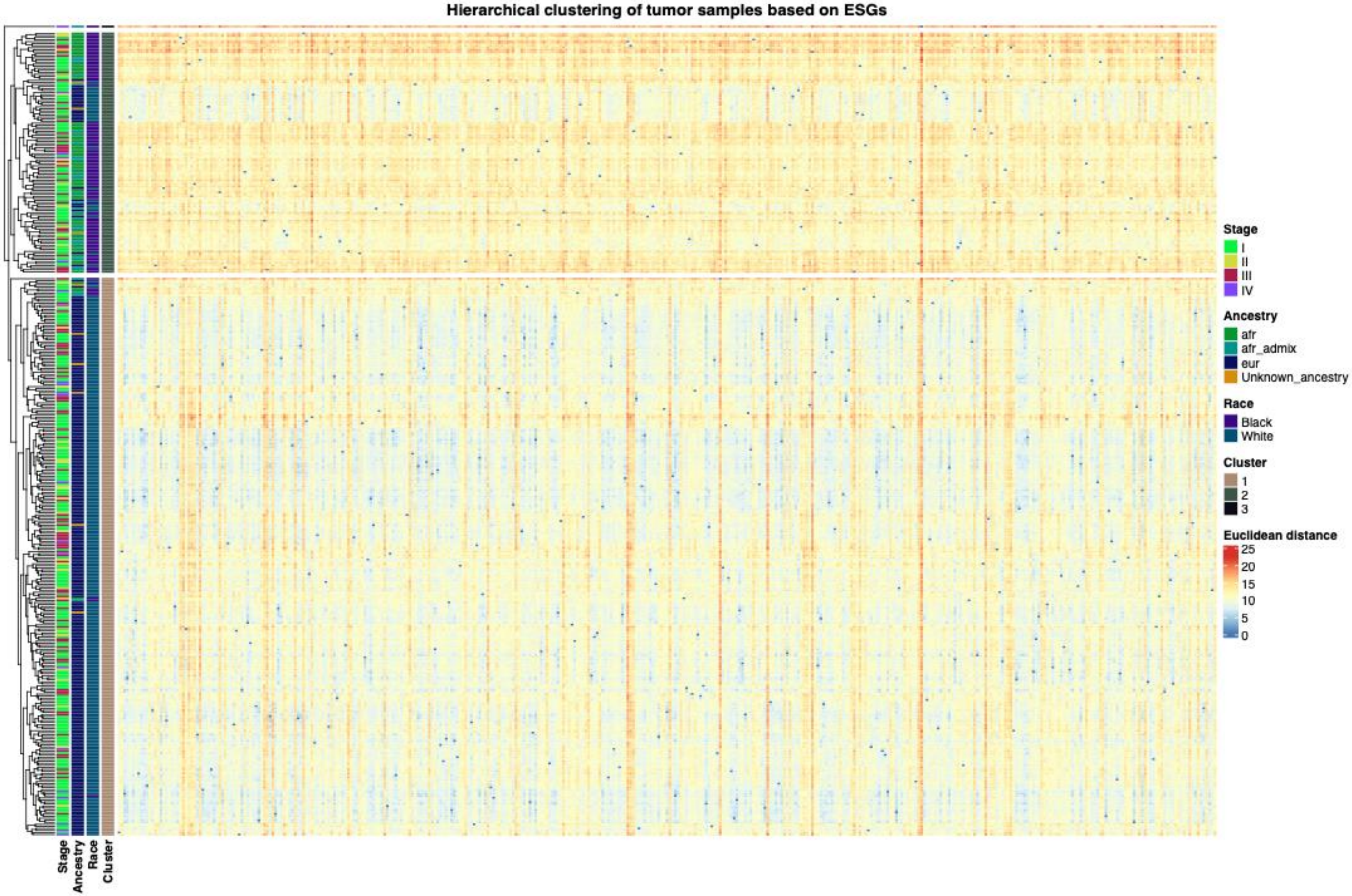

Supplementary Figure S5

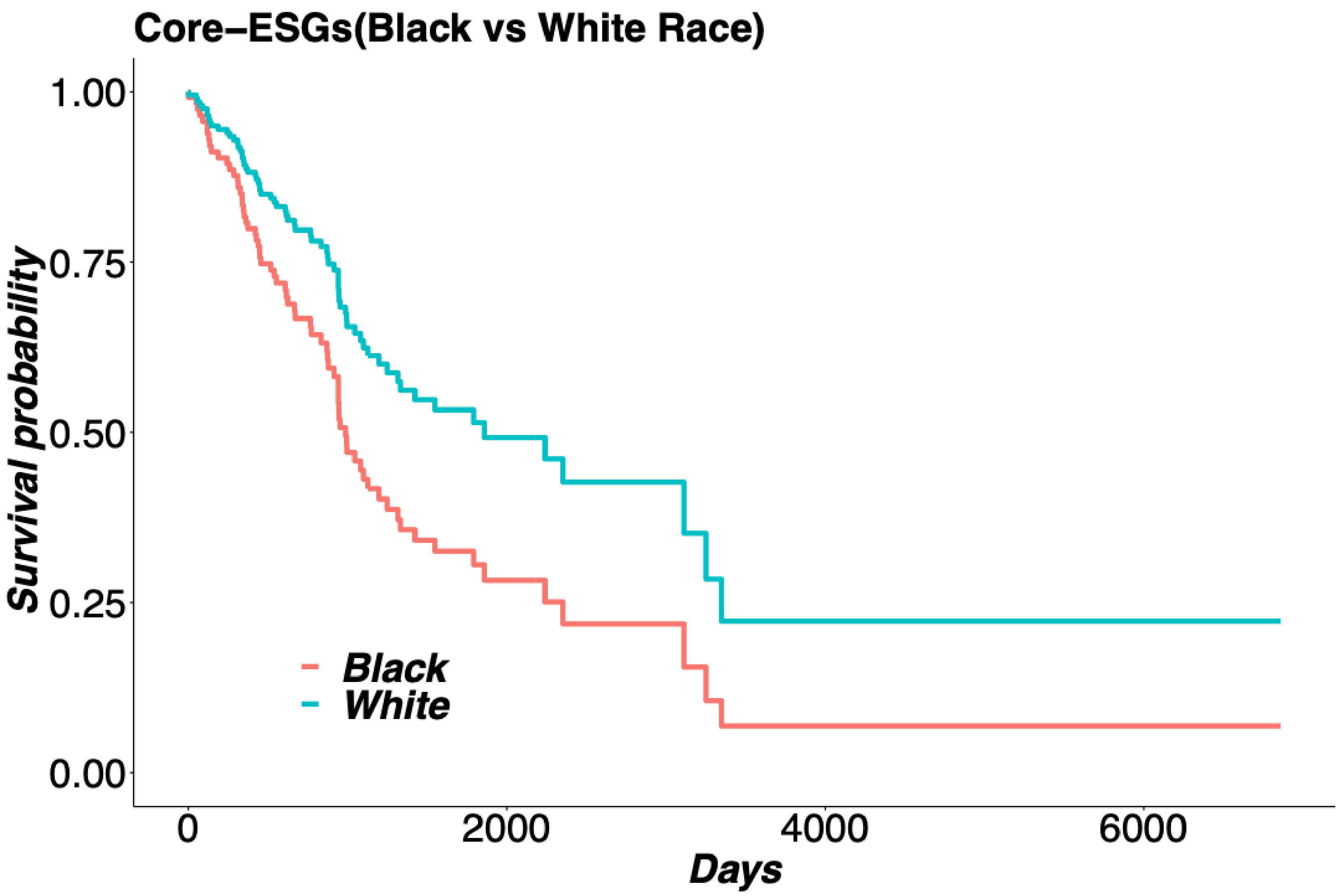

Supplementary Figure S6

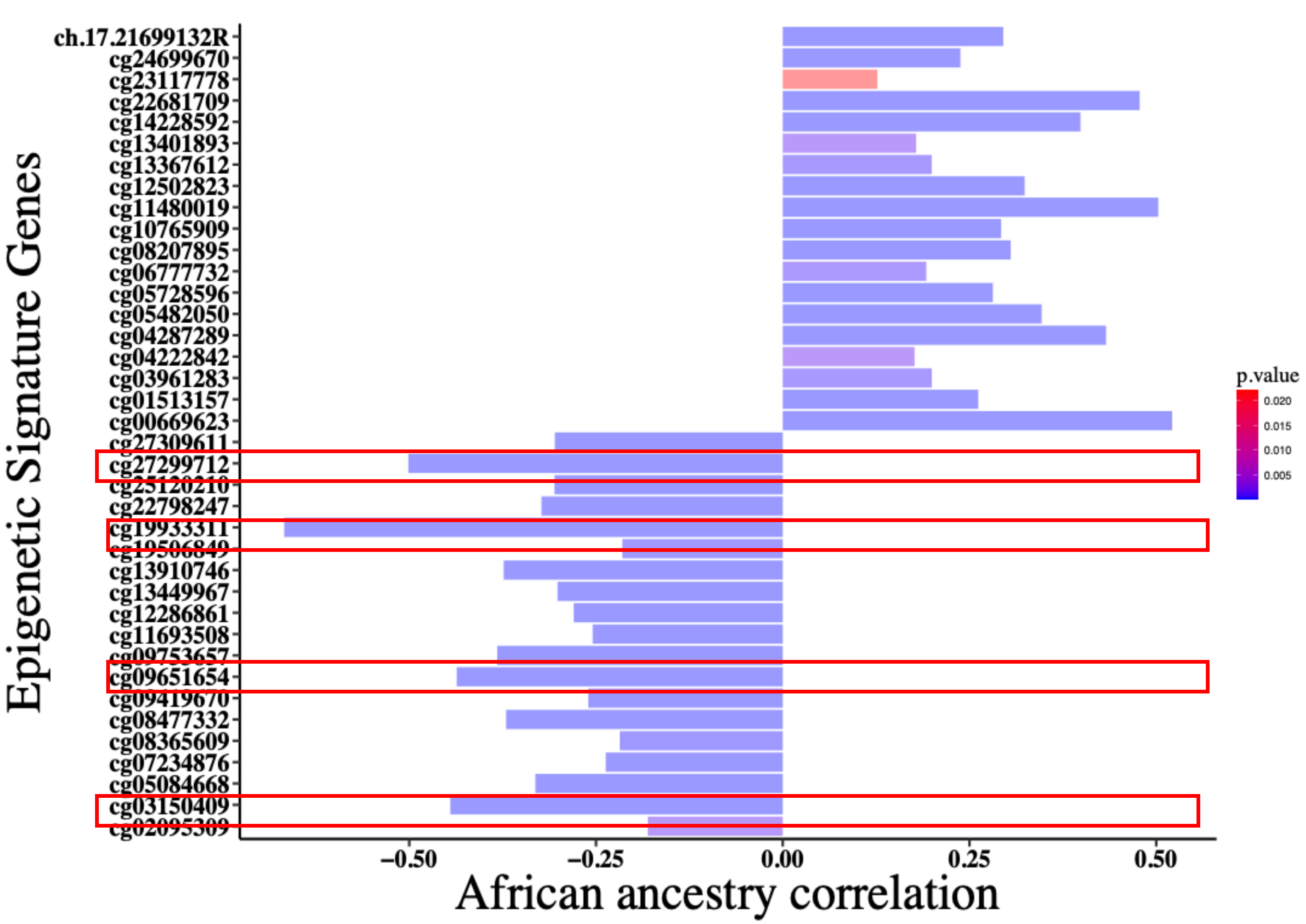

Supplementary Figure S7A

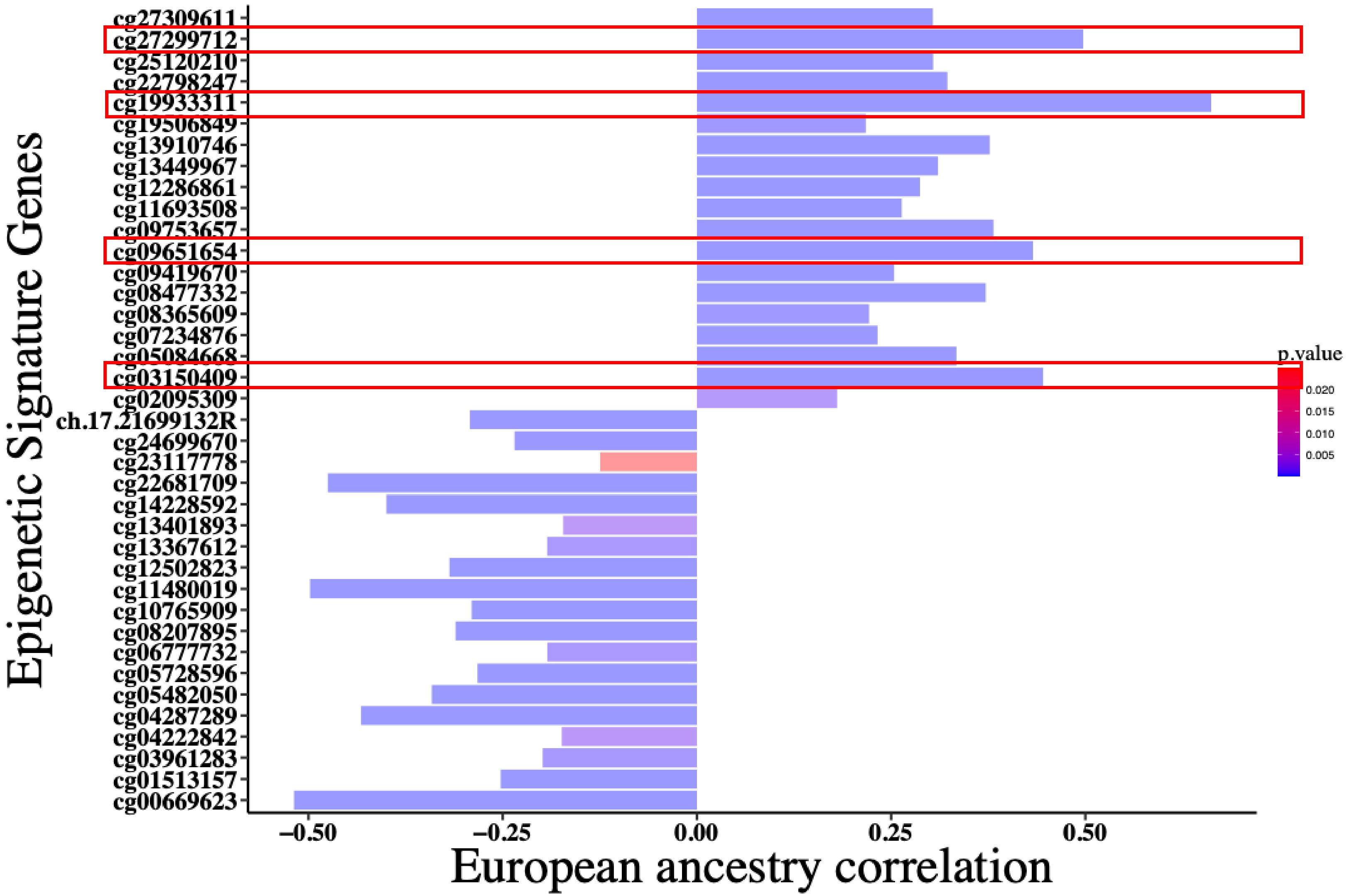

Supplementary Figure S7B
